# Avoiding spiders: Affective context shapes tactile sensitivity during movement

**DOI:** 10.1016/j.isci.2026.117902

**Published:** 2026-10-15

**Authors:** Belkis Ezgi Arikan, Meaghan McManus, Katja Fiehler

**Affiliations:** 1 Department of Neurology, University Hospital Essen, 45147 Essen, Nordrhein-Westfalen, Germany; 2 Center for Translational Neuro- and Behavioral Sciences (C-TNBS), University Hospital Essen, 45147 Essen, Nordrhein-Westfalen, Germany; 3 Experimental Psychology, Justus Liebig University Giessen, 35394 Giessen, Hessen, Germany; 4 Center for Mind, Brain and Behavior (CMBB), University of Marburg and Justus Liebig University Giessen, 35032 Marburg, Hessen, Germany

**Keywords:** tactile suppression, approach-avoidance, emotion, virtual reality

## Abstract

Tactile sensitivity is typically reduced during voluntary movements, a phenomenon known as tactile suppression. While affective context is known to modulate tactile processing, it remains unclear how it influences tactile suppression. In a virtual reality (VR) environment, we investigated tactile sensitivity during movements to avoid threatening (spider) or approach non-threatening (rabbit) stimulus, involving arm flexion or extension and during the limb at rest (baseline). Tactile sensitivity was measured using brief vibrations delivered through the VR controller. Detection thresholds were higher during movement than at baseline, confirming tactile suppression. Both affective context and movement type modulated suppression: avoiding threatening stimulus and extending the arm, each increased suppression. A control experiment using neutral, geometric objects showed similar tactile sensitivity for arm extension and flexion. These findings demonstrate that affective context shapes tactile suppression during movement.

## Highlights

• Affective context shapes tactile sensitivity during movement
• Threatening contexts reduce tactile sensitivity during movement
• Tactile sensitivity differs between flexion and extension across affective contexts

## Graphical abstract

**Figure:**
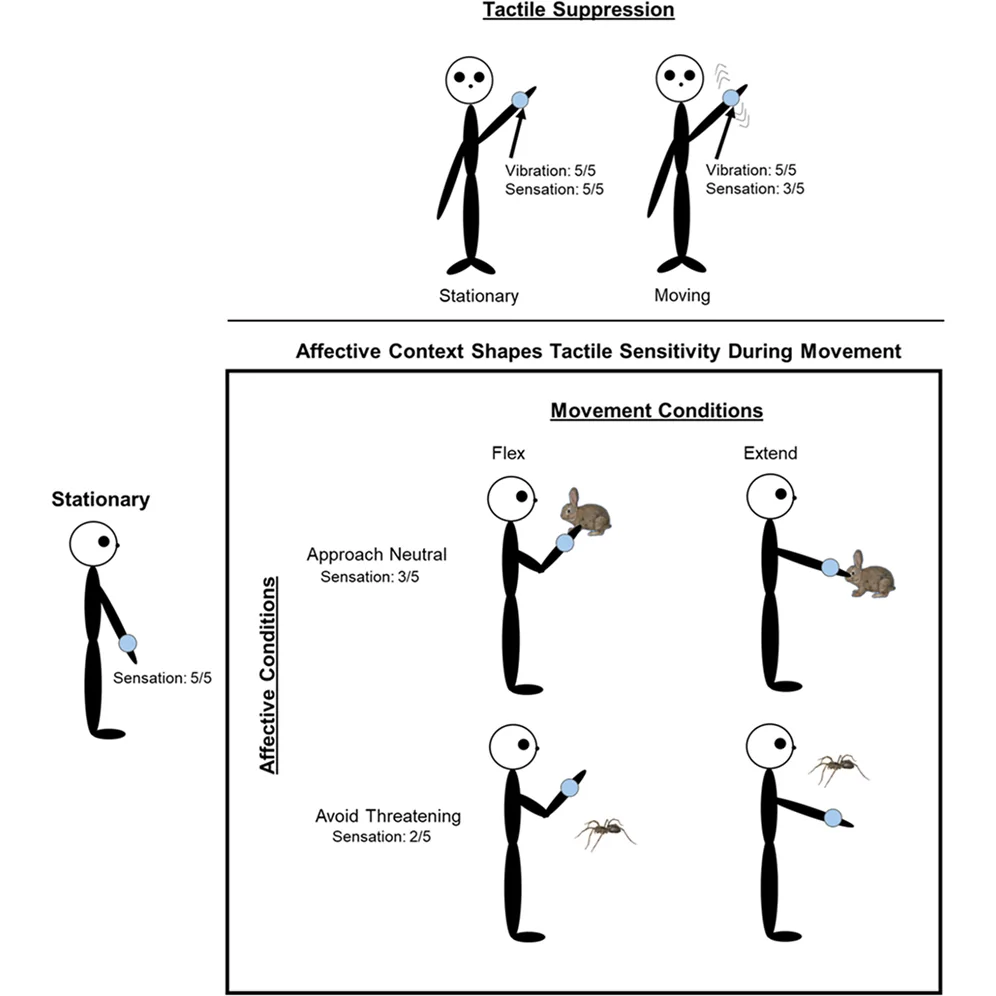

## Teaser

Biological sciences; Neuroscience; Sensory neuroscience

## Introduction

Sensorimotor processes underlying goal-directed movements are essential to navigate our environment. During goal-directed movements, tactile sensitivity on the moving limb is diminished, a phenomenon known as tactile suppression.^1^^,^^2^ Tactile suppression is demonstrated for a range of movements, from those involving fingers^3^^,^^4^^,^^5^ to reaching and pointing,^1^^,^^6^^,^^7^^,^^8^ and whole-body movements.^9^ The underlying mechanism is attributed to internal forward models in which the anticipated bodily states associated with the movement are filtered.^10^^,^^11^ Suppression begins before movement onset, during movement planning, highlighting the role of motor prediction on movement-related tactile sensitivity.^12^^,^^13^

While tactile suppression is an established finding, sensory feedback from the movement is also needed to guide the movement. When tactile feedback is relevant for the movement, i.e., when it is used to monitor and guide the movement, tactile suppression can be reduced.^8^^,^^14^^,^^15^ For instance, tactile suppression is reduced in contexts where precise touch perception is crucial, such as during haptic exploration^15^ or when handling a slippery object.^16^

Tactile feedback is highly relevant in affective contexts, such as during approach and avoidance behaviors. Emotions accompanying these states, for instance fear in the face of threat, are characterized by their valence (appetitive/positive or aversive/negative), and arousal (the intensity of associated physiological and neural activation) and can affect adaptive behavior.^17^^,^^18^ Affective context can prepare the body for imminent threat by modulating tactile processing on the body part, where tactile processing is faster.^19^^,^^20^ Less is known about tactile sensitivity in affective contexts. One study found that fear reduces tactile sensitivity in comparison to neutral emotion.^21^

Similarly, despite the well-established link between emotion and action,^22^^,^^23^^,^^24^^,^^25^^,^^26^^,^^27^^,^^28^ very few studies have investigated the role of movement-related tactile sensitivity in affective contexts. By using microneurography to record from muscle proprioceptive afferents, Ackerley et al.^29^ investigated whether music pieces evoking positive and negative emotions affected proprioception during passive leg movements. Muscle afferent responses increased under emotional music, especially for negative emotions, indicating that motivational states prepare the body for appropriate actions.^30^ To the best of our knowledge, the role of affective context on tactile suppression has not been addressed. The ability to process movement-related tactile feedback can be crucial under threat, as failing to do so may place the organism at risk, while overestimating threat can result in misinterpretation of non-harmful stimuli as harmful, as seen in phobias.^31^^,^^32^ Here, we tested the assumption that changes in bodily state, induced by the affective context, can modulate tactile sensitivity on the moving limb. More specifically, negative affective context evoking avoidance responses may prioritize motor prediction over feedback signals in order to efficientlywithdraw from potential threat.

In addition, we aimed to address the role of motor predispositions (e.g., extending the arm for approaching) on tactile suppression. Previous findings indicate that motivationally salient events elicit motor behaviors that facilitate certain responses, such as faster approach toward pleasant stimuli by pulling them closer and faster avoidance of unpleasant stimuli by pushing them away.^33^^,^^34^ These findings suggest predispositions like arm flexion for approach and arm extension for avoidance responses. However, more recent studies indicate that humans flexibly adapt their motor responses based on their intended goal,^35^^,^^36^^,^^37^^,^^38^ but see Eder et al.^39^ Our second aim was to address the role of these predispositions on tactile suppression under threat.

In this study, we investigated tactile suppression in a virtual reality (VR) environment as participants interacted with threatening and non-threatening stimuli that were associated with actions to avoid or approach them, respectively. We chose this specific mapping to study ecologically valid action tendencies, namely avoiding threatening and approaching non-threatening stimuli. We also examined if motor predispositions that accompany avoidance/approach responses further modulate tactile suppression. VR provides a more naturalistic context that closely mimics real-world interactions, thereby enhancing ecological validity while maintaining experimental control. Participants encountered a virtual spider (a threatening stimulus), or a rabbit (a non-threatening stimulus). They were instructed to avoid the spider or approach the rabbit by an arm extension or flexion during which tactile sensitivity on their moving hand was probed with vibrations of different intensities (or no vibration). In each trial, they reported the presence or absence of the vibration. A control condition in which the participants held their arms static in a virtual sphere served as a baseline. We hypothesized that tactile suppression would be modulated by the relevance of motor prediction and feedback signals such that avoiding the threatening stimulus would result in larger tactile suppression than approaching the non-threatening stimulus as motor predictions gain more relevance in the former case.

## Results

### Experiment 1

Participants performed dynamic arm movements (extension or flexion) where they approached a non-threatening rabbit or avoided a threatening spider stimulus. They simultaneously performed a tactile detection task where they judged if a vibration was delivered via the VR controller (see Figure 1 for an overview of the experimental setup and design, and Figure 2 for timeline of an experimental trial). We investigated whether threatening and non-threatening stimuli differently affected tactile detection during movement and if different arm movements influenced this. Then, we tested the effect of movement on tactile detection.

**Figure 1:**
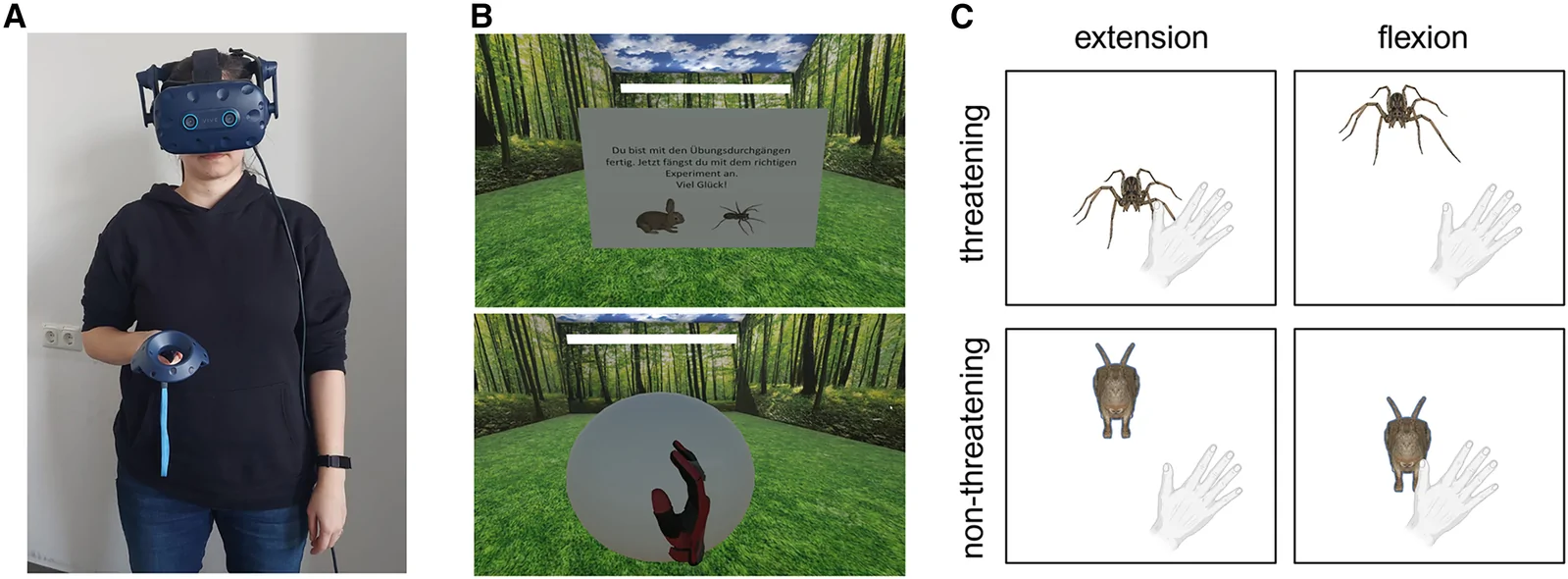
Experimental setup and visual environment in experiment 1 (A) Example participant. (B) The virtual forest environment. The top figure shows one of the instruction slides presented in the virtual environment. The bottom figure shows the right hand of the participant in the virtual environment along with the trial starter sphere. The images are screenshots of the virtual environment. (C) Experimental design. Affective stimuli (threatening spider vs. non-threatening rabbit) and movement type (extension vs. flexion). See Video S1 for an example of experimental trials. Created in BioRender. Arikan, B. E. (2026) https://BioRender.com/d7c8ch9.

**Figure 2:**
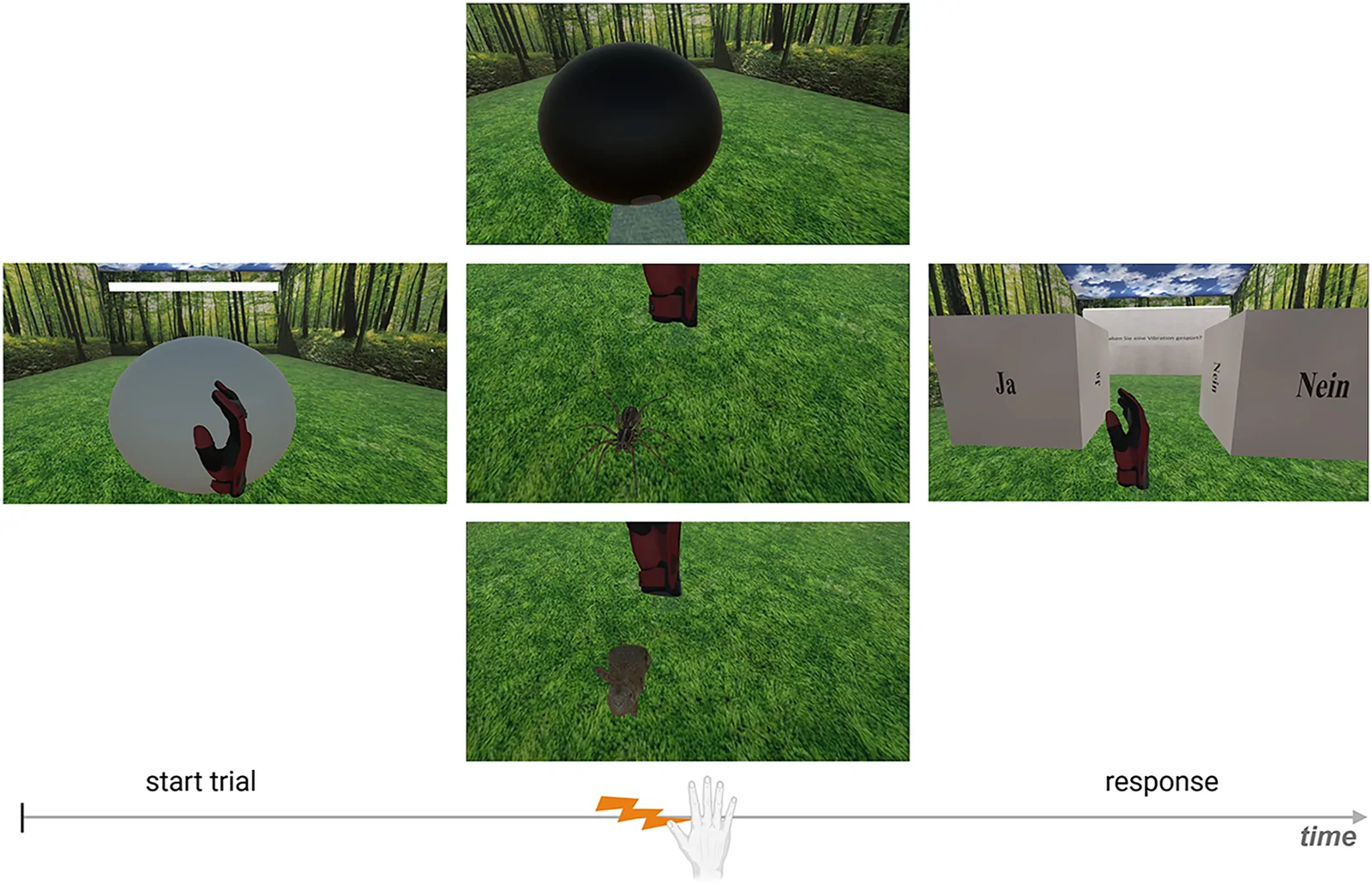
Timeline of an experimental trial in experiment 1 The images are screenshots of the virtual environment. In the beginning, a gray sphere indicates the start of a trial. The participants were then presented with one of the three experimental objects: a black sphere corresponded to the baseline condition and instructed participants to keep their virtual hand within the sphere and not exert any movement. The spider (middle) corresponded to the threatening stimulus and instructed participants to keep their virtual hand away from the spider. The rabbit (bottom) corresponded to the non-threatening stimulus and instructed participants to move toward the rabbit. Upon occurrence of the spider or the rabbit, the participants performed an extension or a flexion movement away from or toward the stimuli, respectively. During this movement or in the baseline condition the participants might receive vibration to their hand. They responded if they detected any vibration once the experimental objects disappeared. Created in BioRender. Arikan, B. E. (2026) https://BioRender.com/3531bv4.

### Tactile perception: Decreased tactile sensitivity during movement

Figures 3 and S1 depict psychometric fits in each condition from a representative participant and from each participant, respectively (see Table S1 for the individual thresholds and deviance scores). We tested if tactile suppression was present in all movement conditions (extension and flexion away from and toward the spider and the rabbit, respectively), compared to the baseline condition without arm movement. A linear mixed model (LMM) on detection thresholds with condition (baseline, spider-extend, spider-flex, rabbit-extend, and rabbit-flex) as fixed effect of interest, condition randomization as fixed effect of no interest, and participant as random effect resulted in a main effect of condition (see Table 1). Post hoc pairwise comparisons revealed that tactile detection was significantly lower in all movement conditions compared to the baseline: baseline vs. spider-extend (95% confidence interval [CI]: 0.03, 0.08): d = 0.96, *p* < 0.001; baseline vs. spider-flex (95% CI: 0.03, 0.08): *d* = 0.83, *p* < 0.001; baseline (95% CI: 0.002, 0.05) vs. rabbit-extend (95% CI: 0.03, 0.08): *d* = 0.87, *p* < 0.001; baseline vs. rabbit-flex (95% CI: 0.02, 0.07): *d* = 0.59, *p* < 0.001 (see Table S2). These results confirm tactile suppression in all conditions with movement.

**Figure 3:**
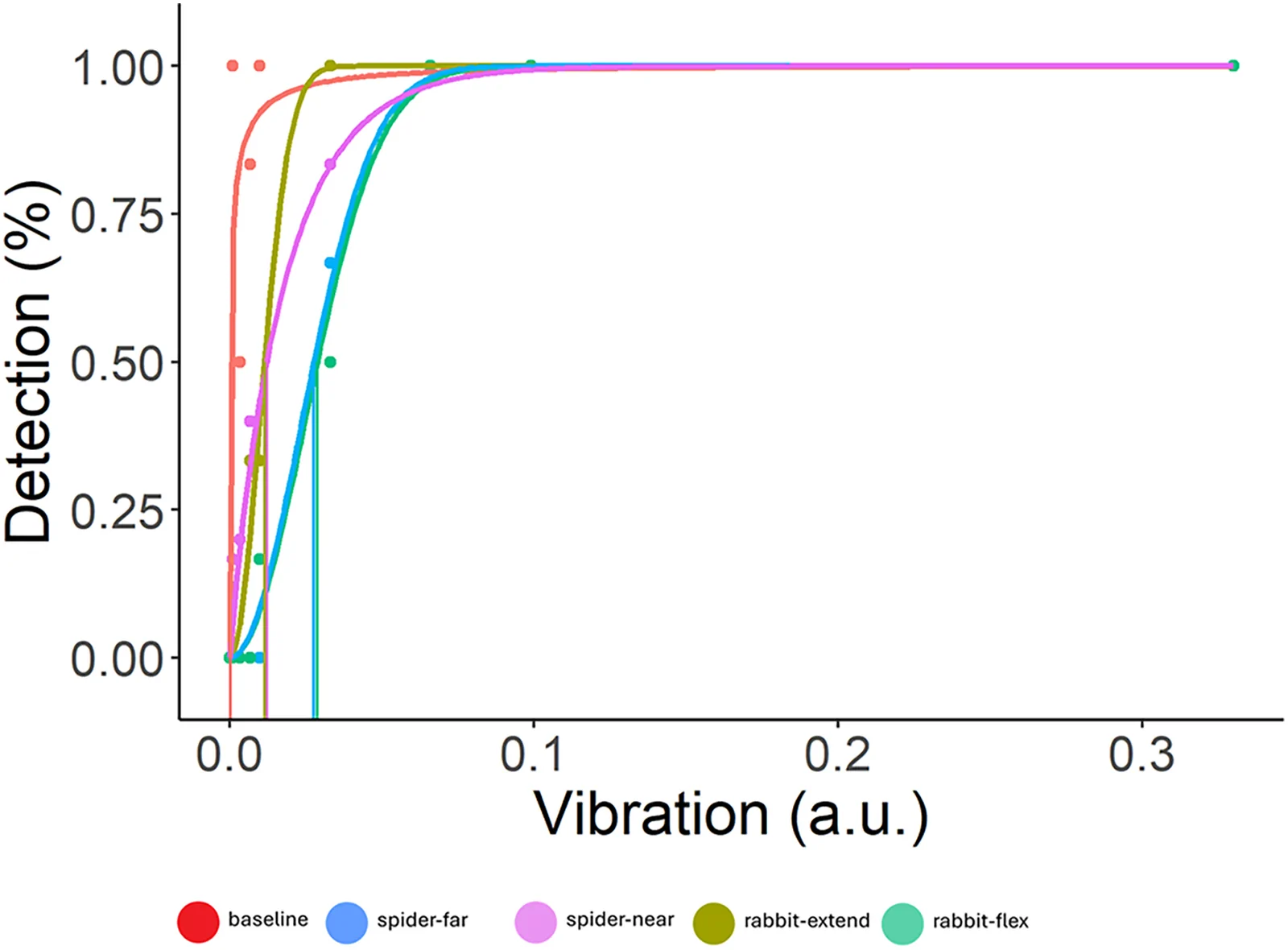
Psychometric functions from a sample participant depicting tactile detection as a function of vibration intensity across conditions In line with Fechner’s law, the vibrations used in this experiment follow a log scale (plus the no vibration condition). When determining the individual thresholds we included the no-vibration condition and could not plot the data on a logged scale (see Figure S1 for individual fits and Figure S7 for example of an individual participant’s data plotted on a logarithmic scale, which illustrates the fit of the psychometric function in detail); a.u., arbitrary units.

**Table 1:** LMM testing tactile sensitivity during movement

	*SS*	*MS*	*dfs*	*F*	*p*
Movement	0.023	0.006	4, 168	12.59	<0.001
Condition randomization	0.001	0.001	1, 41	2.85	0.099

Statistics are reported for each item with sum of squares (*SS*), mean squares (*MS*), degrees of freedom (*df*s), F value (*F*), and *p* value (*p*).

### Response bias: Similar false alarm rates across conditions

Overall, false alarm rates were relatively low (mean ± standard deviation, SD): baseline = 0.05 ± 0.14, spider-extend = 0.02 ± 0.11, spider-flex = 0.05 ± 0.13, rabbit-extend = 0.04 ± 0.11, rabbit-flex = 0.05 ± 0.14. An LMM on false alarm rates with conditions (baseline, spider-extend, spider-flex, rabbit-extend, and rabbit-flex) as fixed effect of interest, condition randomization as fixed effect of no interest, and participant as random effect did not reveal any significant differences between the conditions; *F*(4, 168) = 1.03, *p* = 0.40 (sum of squares [SS] = 0.024, mean of squares [MS] = 0.006). Condition order was also not significant: *F*(1, 41) = 1.24, *p* = 0.27 (SS = 0.007, MS = 0.007).

### Movement speed: Faster movements when interacting with the threatening spider compared to the non-threatening rabbit stimulus

An LMM on the movement speed testing the effect of affective stimulus (threatening spider vs. non-threatening rabbit) and movement type (extend vs. flex) resulted in a main effect of affective stimulus, *F*(1, 126) = 14.01, *p* < 0.001 (SS = 0.12, MS = 0.12), revealing faster movements for the threatening spider (95% CI: 0.39, 0.46) than for the non-threatening rabbit (95% CI: 0.34, 0.41) stimulus. All other factors were non-significant. The results can be seen in Figure 4B.

**Figure 4:**
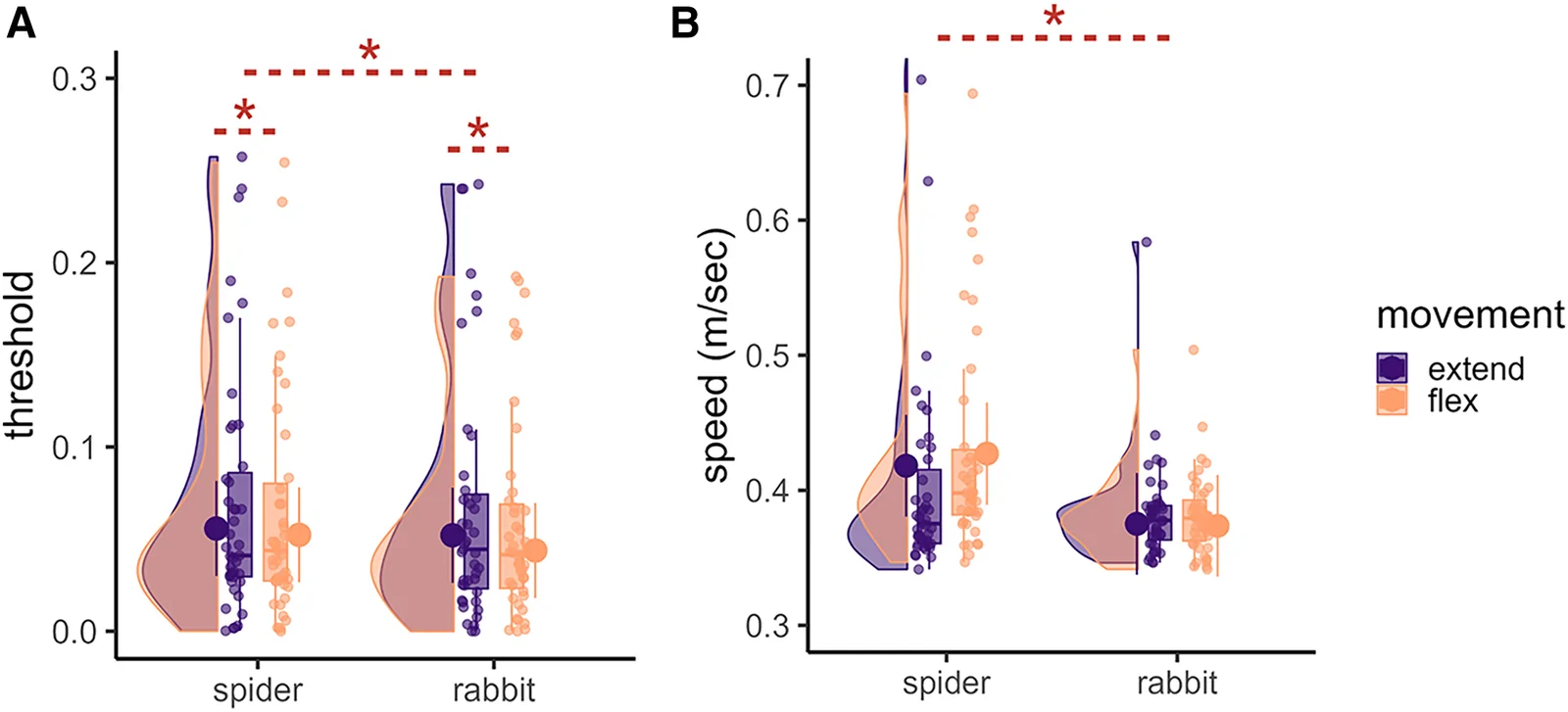
Raincloud plots showing tactile detection thresholds and movement speed as a function of affective stimulus and movement type (A) Tactile detection thresholds and (B) movement speed. For visualization purpose, one data point from the spider-extend condition (speed variable) was excluded from the plot (speed >0.7 m/s). Dots represent individual participant data. Boxplots display the median and interquartile range, with whiskers extending to ±1.5 times the interquartile range. Large dots indicate estimated marginal means from the linear mixed models, with vertical lines showing 95% confidence intervals (*n* = 43 spider-extend, *n* = 43 spider-flex, *n* = 43 rabbit-extend, and *n* = 43 rabbit-flex).

### Tactile perception: Decreased tactile sensitivity for threatening spider and extension movements

We tested whether tactile suppression was modulated by affective stimulus and movement type. For this, we conducted an LMM on detection thresholds in the movement conditions as a function of affective stimulus (threatening spider vs. non-threatening rabbit) and movement type (extension vs. flexion), condition randomization as fixed effect of no interest, movement speed as covariate, and participant as random effect. The results can be seen in Table 2 and Figure 4A. There was a main effect of affective stimulus, revealing stronger suppression for the threatening spider (95% CI: 0.03, 0.08) in comparison to the non-threatening rabbit stimulus (95% CI: 0.02, 0.07), *d* = 0.37. We also found the main effect of movement type whereby extension movements (95% CI: 0.03, 0.08) led to larger tactile suppression than flexion movements (95% CI: 0.02, 0.07), *d* = 0.37. All other factors were non-significant.

**Table 2:** LMM testing affective stimuli and movement type on tactile suppression

	*SS*	*MS*	*dfs*	*F*	*P*
Affective stimulus	0.001	0.001	1, 125.38	4.23	0.04
Movement type	0.002	0.002	1, 124.84	4.34	0.04
Affective stimulus × movement type	0.0003	0.0003	1, 124.84	0.76	0.39
Condition randomization	0.001	0.001	1, 40.81	2.89	0.1
Movement speed (covariate)	0.001	0.001	1, 130.40	2.34	0.13

Statistics are reported for each item with sum of squares (*SS*), mean squares (*MS*), degrees of freedom (*df*s), F value (*F*), and *p* value (*p*).

Together, these results confirm the well-established effect of tactile suppression during movement. In addition, they indicate that affective stimuli and movement type have different influences on tactile suppression.

### Self-report measures: Spider stimulus is a less pleasant cue compared to the rabbit stimulus

Figure 5 and Table S3 show ratings of valence, arousal, anxiety, and disgust for spider and rabbit stimuli. The LMMs investigating the effect of affective stimulus on the valence, arousal, anxiety, and disgust ratings showed a significant effect of affective stimulus on all the ratings. These results show that the spider stimulus was perceived as less pleasant, more arousing, anxiety-provoking, and disgusting than the rabbit stimulus.

**Figure 5:**
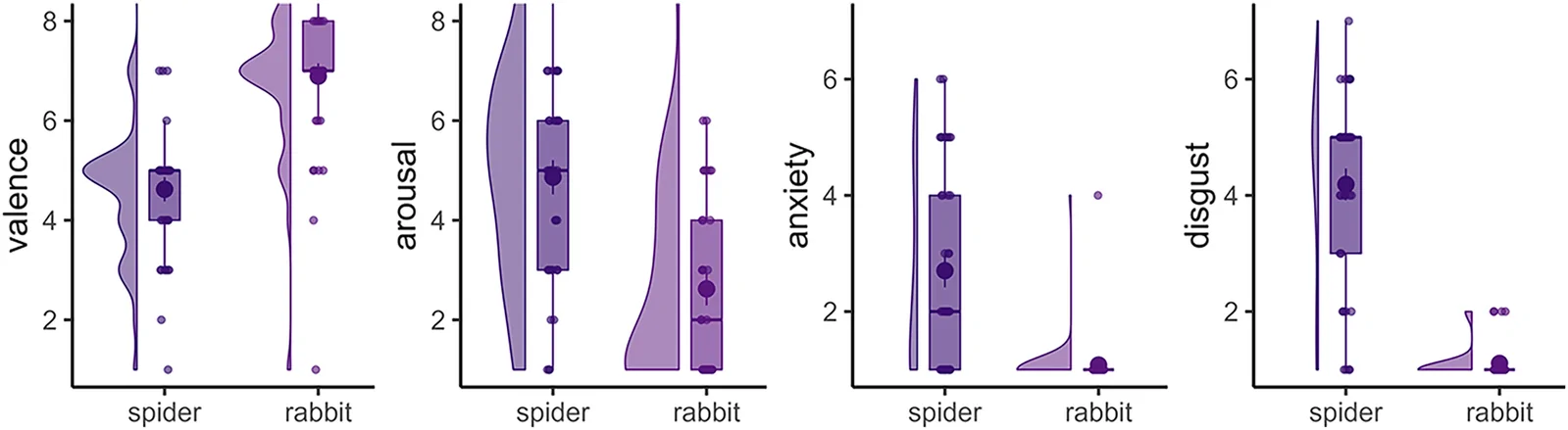
Raincloud plots showing valence, arousal, anxiety, and disgust ratings as a function of affective stimulus Dots represent individual participant data. Boxplots display the median and interquartile range, with whiskers extending to ±1.5 times the interquartile range. Large dots indicate estimated marginal means from the linear mixed models, with vertical lines showing 95% confidence intervals (*n* = 37 spider and *n* = 37 rabbit).

### Correlations between tactile suppression and self-report measures

None of the correlations between valence, arousal, anxiety, and disgust ratings, and detection thresholds reached significance, nor did the correlations between state- and trait-anxiety measures and detection thresholds or cognitive task load (see Figures S2–S4; also see Table S4 for NASA task load ratings).

### Interim discussion

Together, the results suggest additive effects of affective context and motor predisposition on tactile sensitivity during movement. First, the threatening spider stimulus resulted in larger tactile suppression than the non-threatening rabbit stimulus, indicating the importance of motor predictions during threat avoidance. Second, we found larger tactile suppression for arm extension compared to flexion. Since the speed of extension and flexion movements did not differ, we could rule out speed as a likely explanation for the difference. A potential explanation for this finding could be related to movement kinematics. As can be seen in Figure 6 (see STAR Methods), the participants appear to successfully move to different locations during extension and flexion movements for both the spider and rabbit stimuli. However, we considered the possibility that extension movements may be more demanding in terms of movement kinematics,^40^ resulting in larger suppression. To test this, we conducted a control experiment in which we tested if extension and flexion toward neutral, inanimate stimuli led to differences in tactile suppression.

**Figure 6:**
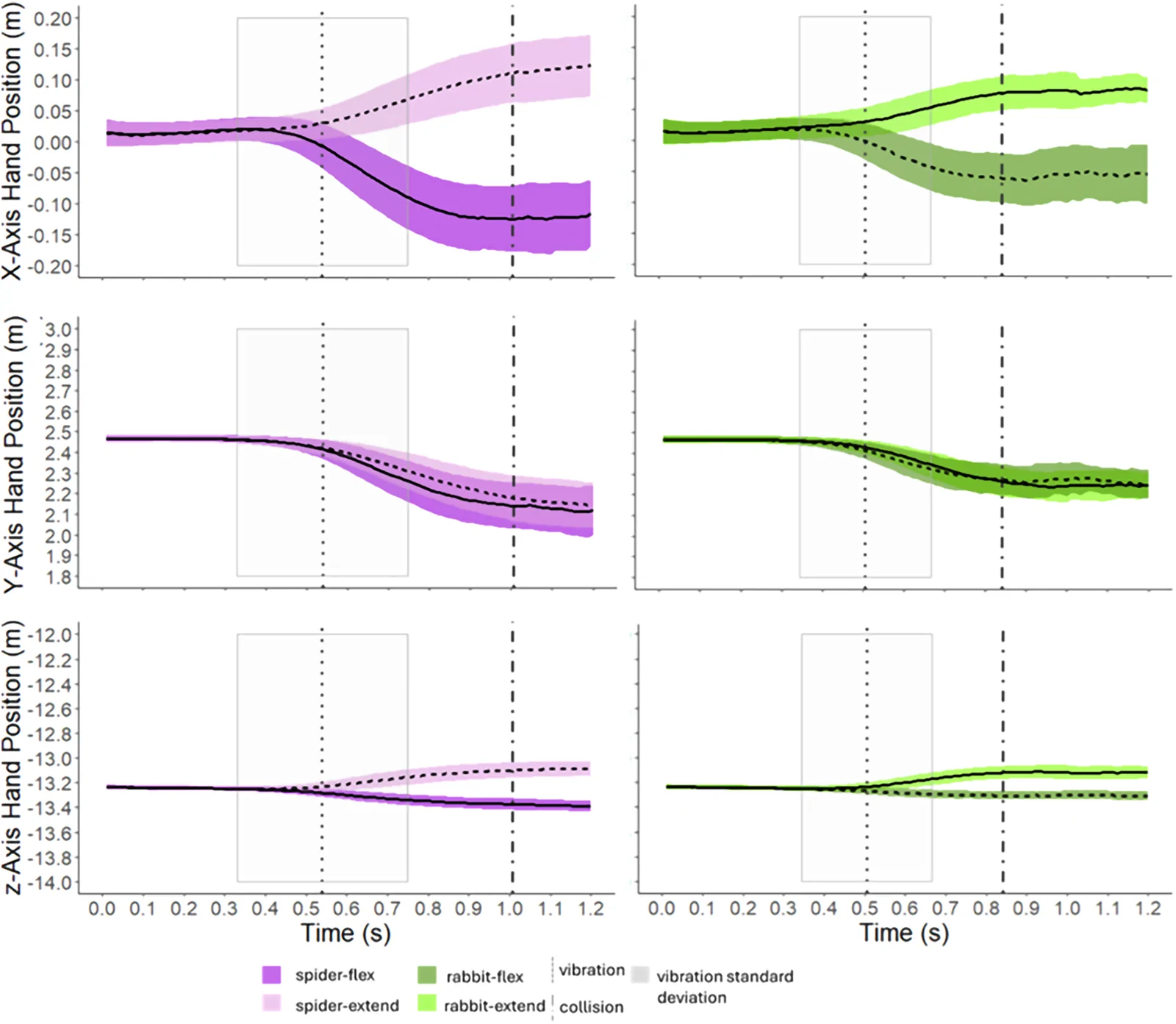
Average hand position for each movement condition along each axis in experiment 1 For the plotted trajectories as the hand moved along the *x* axis (left and right movements, top row), the higher the line, the more to the right the participant’s hand was moving. For the plotted trajectories as the hand moved along the *y* axis (up and down movements, middle row), the higher the line, the higher the hand’s position in the virtual environment. For the plotted trajectories as the hand moved along the *z* axis (forward and back, bottom row), the higher the line, the more forward the participants’ hands were moving. The horizontal shaded areas in the figures represent the ±standard deviation around the mean hand position across all participants and trials for a given condition. The median time at which the controller could have vibrated and the collision occurred are indicated in the figures as the dotted line and the dot dash line, respectively. The gray vertical shaded area corresponds to the standard deviation around the median time the controller vibrated.

### Experiment 2

#### *Tactile perception: Decreased tactile sensitivity during movement but no differences between movement conditions*

Participants performed dynamic arm movements (extension or flexion) towards a virtual box or and were asked to detect whether or not a vibration was delivered via the VR controller (see Figure 7 for timeline of an experimental trial). Figure 8 shows psychometric fits from a representative participant (see Figure S5 for individual fits and Table S5 for the individual thresholds and deviance scores). LMM on detection thresholds with movement (extend, flex, and baseline) as fixed effect of interest and participant as random effect resulted in the main effect of movement; *F*(2, 61.88) = 24.82, *p* < 0.001 (SS = 0.01, MS = 0.005). Post hoc pairwise comparisons revealed that tactile detection was significantly lower in both movement conditions compared to the baseline: baseline (95% CI: 0.002, 0.02) vs. extend (95% CI: 0.02, 0.04): estimate = −0.02, *t* = −5.65, *d* = 1.42, *p* < 0.001; baseline vs. flex (95% CI: 0.03, 0.04): estimate = −0.02, *t* = −6.52, *d* = 1.64, *p* < 0.001 (see also Table S6). The difference between extend and flex did not reach significance; estimate = −0.003, *t* = −0.82, *p* > 0.1. Second, LMM on thresholds with the fixed effect movement (extend vs. flex), speed as covariate, and participant as random effect resulted in a non-significant effect of movement *F*(1, 31.02) = 0.16, *p* = 0.69 (SS = 0.00002, MS = 0.00002). The effect of speed was significant; *F*(1, 50.70) = 9.17, *p* = 0.004 (SS = 0.001, MS = 0.001). The Bayesian paired-samples *t* test comparing detection thresholds for the extend and flex conditions yielded a Bayes factor of BF10 = 0.22, providing moderate evidence in favor of the null hypothesis. Together, these results show similar tactile suppression across arm extension and flexion when movements were directed toward neutral, static stimuli (see Figure 9 for an overview of hand position across movement conditions). However, suppression did not seem to differ between arm extension and flexion.

**Figure 7:**
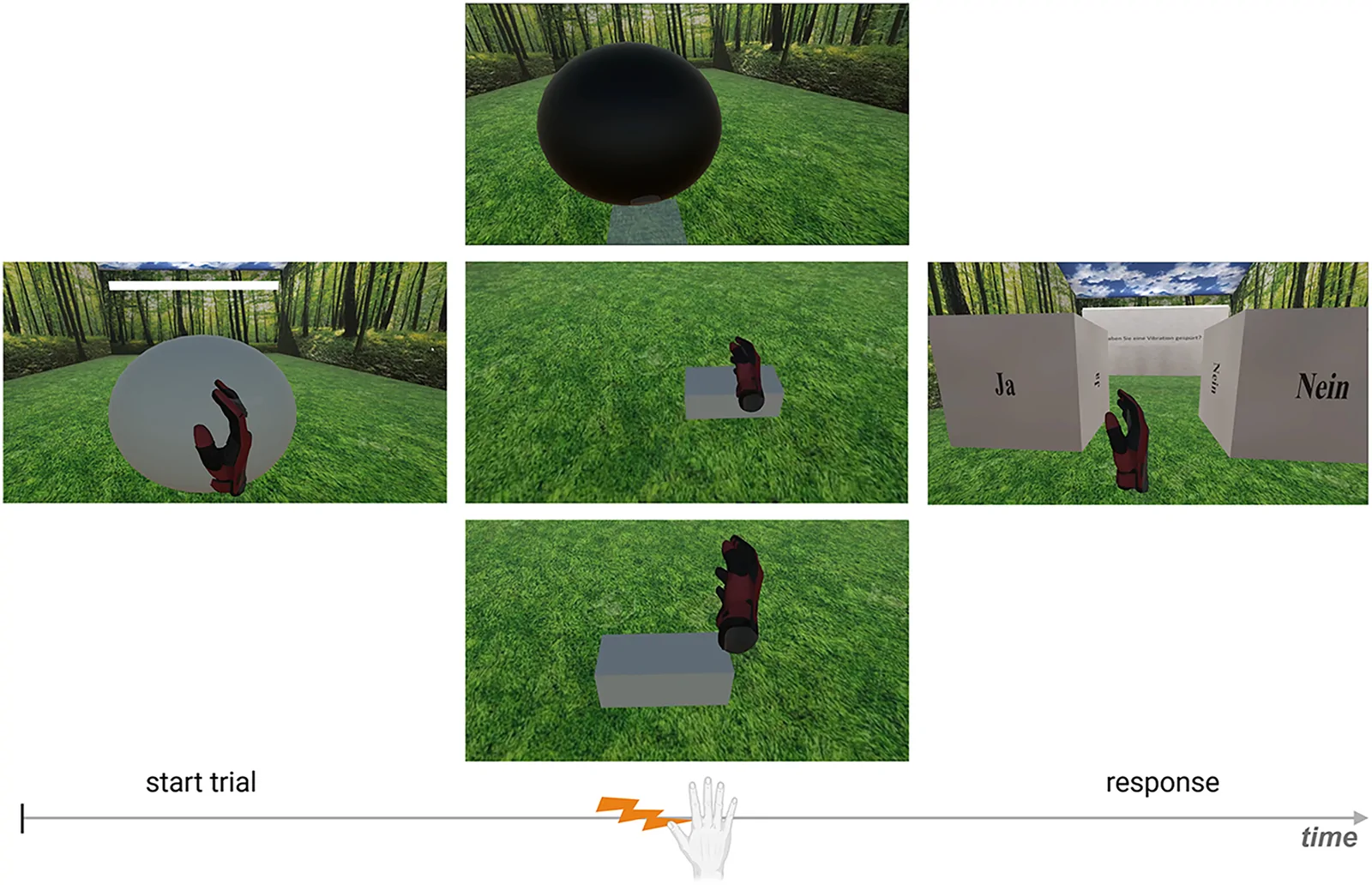
Timeline of an experimental trial in experiment 2 The images are screenshots of the virtual environment. In the beginning, a gray sphere indicates the start of a trial. The participants were then presented with one of the three virtual objects: a black sphere corresponded to the baseline condition and instructed participants to keep their virtual hand within the sphere and not exert any movement. The extension condition (middle) instructed participants to perform an extension movement. The flexion condition (bottom) instructed participants to perform a flexion movement. During the movement or the baseline conditions, participants could receive vibration to their hand delivered via the VR controller. After the trial they had to respond if they felt the vibration or not. Created in BioRender. Arikan, B. E. (2026) https://BioRender.com/gqir1dp.

**Figure 8:**
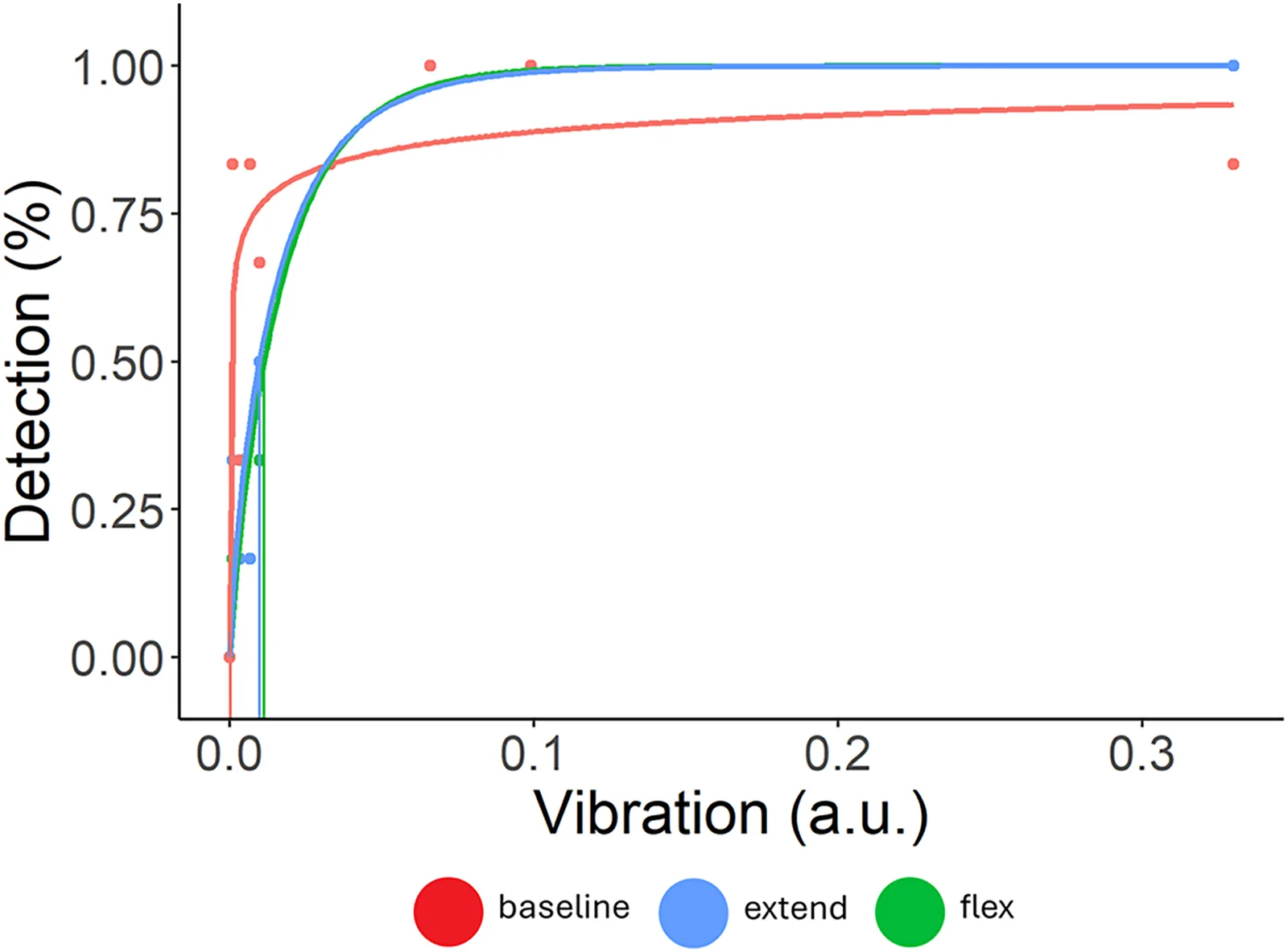
Psychometric functions from a sample participant depicting tactile detection as a function of vibration intensity across conditions In line with Fechner’s law, the vibrations used in this experiment follow a log scale (plus the no vibration condition). When determining the individual thresholds we included the no-vibration condition and so could not plot the data on a logged scale (see Figure S5 for individual fits); a.u., arbitrary units.

**Figure 9:**
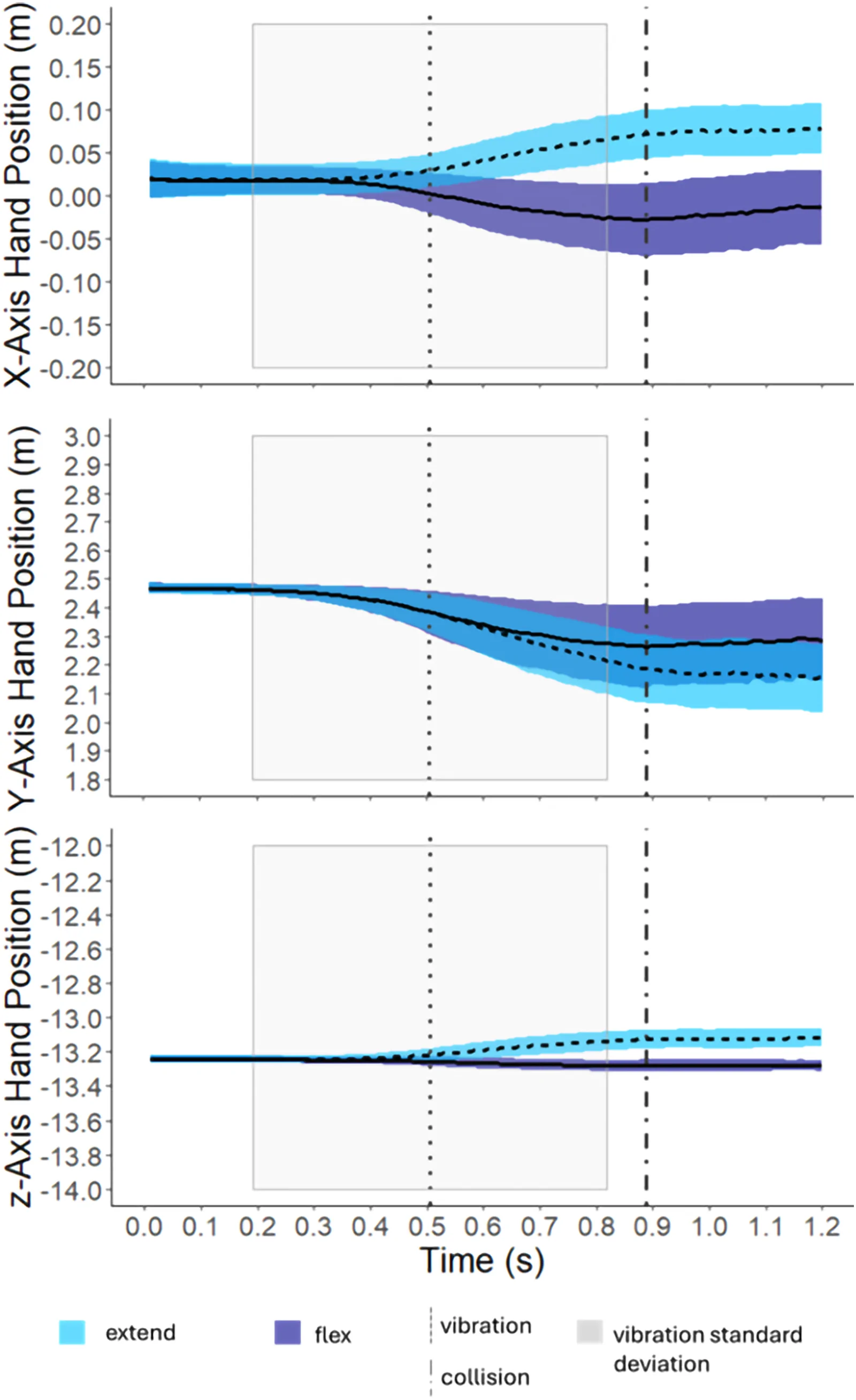
Average hand position for each movement condition along each axis in experiment 2 For the plotted trajectories as the hand moved along the *x* axis (left and right movements, top row), the higher the line, the more to the right the participant’s hand was moving. For the plotted trajectories as the hand moved along the *y* axis (up and down movements, middle row), the higher the line, the higher the participant’s hand position in the virtual environment. For the plotted trajectories as the hand moved along the z axis (forward and back, bottom row), the higher the line, the more forward the participant’s hand was moving. The horizontal shaded areas in the figures represent the ± standard deviation around the mean hand position across all participants and trials for a given condition. The median time at which the controller could have vibrated and the collision occurred are indicated in the figures as the dotted line and the dot dash line, respectively. The gray vertical shaded area corresponds to the standard deviation around the median time the controller vibrated.

### Response bias: Similar false alarm rates across conditions

Similar to experiment 1, false alarm rates were relatively low (mean ± SD): baseline = 0.02 ± 0.07, extend = 0.07 ± 0.13, flex = 0.07 ± 0.11. LMM on false alarm rates with conditions (baseline, extend, and flex) as fixed effect of interest and participant as random effect revealed a marginally significant effect of condition; *F*(2, 68) = 3.14, *p* = 0.05 (SS = 0.05, MS = 0.03). However, post hoc pairwise comparisons were not significant after correction for multiple comparisons (all *p* > 0.05, see Table S7 for contrasts).

### Correlations between tactile suppression and self-report measures

None of the correlations between detection thresholds and cognitive task load reached significance (see Figure S6; also see Table S4 for NASA task load ratings).

## Discussion

Here, we investigated the role of affective context and motor predispositions on tactile suppression. We used VR to increase the feeling of presence and immersion that can facilitate affective responses, such as when playing a fearful game.^41^ Affective ratings for the spider and rabbit stimuli indicated successful induction of affective context: participants rated the former as less pleasant and more arousing, anxiety-provoking, and disgusting than the latter. Our results showed larger tactile suppression when avoiding the threatening spider stimulus than when approaching the non-threatening rabbit stimulus. In addition, extension movements led to greater tactile suppression, though the movement type did not interact with the affective stimulus. To rule out the possibility that movement kinematics inherent to extending and flexing the arm differ in terms of tactile suppression, we ran a control experiment in which we quantified tactile suppression for arm extension and flexion to neutral geometric objects. The results showed no systematic differences in tactile suppression between these movements. Importantly, response bias could be ruled out as the potentially modulating factor of tactile sensitivity in both experiments, as we found similar false alarm rates across conditions. Together, our results indicate additive effects of affective context and motor predispositions in tactile suppression.

Exposure to potential threat prepares the body for action^25^^,^^26^ and can potentially impact tactile sensitivity associated with the action. Here, we asked whether affective context influences movement-related tactile sensitivity, i.e., tactile suppression. As tactile suppression depends on the relevance of motor prediction and sensory feedback to the movement,^8^^,^^14^^,^^15^^,^^16^ we asked if changes in bodily states induced by the affective context modulate tactile sensitivity on the moving limb. We found that movements aimed at avoiding a threatening spider stimulus resulted in larger tactile suppression than similar movements aimed at approaching a non-threatening rabbit stimulus, after taking movement speed into account. These findings are consistent with the possibility that negative affective context leads to increased prioritization of motor prediction over sensory feedback, potentially enabling efficient withdrawal from potential threat. It remains possible that the findings could be due to changes in sensory weighting; how to disentangle the two is unclear. In addition, our findings underline the role of behavioral flexibility rather than motor predispositions in modulating tactile suppression in affective contexts.^36^^,^^42^

It has been postulated that affective context can boost motor predispositions (e.g., arm flexion during threat avoidance).^33^^,^^34^^,^^43^ Conversely, movement goals can be more important in modulating behavior than motor predispositions.^35^^,^^36^^,^^42^ Our results did not support the existence of an interplay between affective context and motor predisposition in modulating tactile suppression. On the other hand, we found larger tactile suppression for arm extension than flexion. To rule out the possibility that suppression essentially differs between these movements in terms of their kinematic properties, we conducted a control experiment in which extension and flexion movements targeted neutral geometric objects. We found similar levels of suppression during these movements. One possibility is that some of the differences in tactile suppression between extension and flexion movements in experiment 1 may relate to higher-order properties of the stimuli used in that experiment, which were not present in experiment 2. Perhaps any animacy attributed to these objects could enhance visuotactile interactions in the space directly surrounding the body and within the reachable space, also known as the peripersonal space. The importance of motivational salience on peripersonal space has been demonstrated in animals^44^^,^^45^ and humans.^19^^,^^46^ Peripersonal space is influenced by many factors including direction of the bodily motion.^47^ It could be that movements toward the body may have enhanced the processing of feedback signals in the near space more so than in the far space, as this more immediate space potentially requires faster responses in interacting with the environment.^48^ The reduced tactile suppression during flexion movements was independent of the approach/avoid response in experiment 1, suggesting that movement-related effects could potentially depend on how action goals are represented in near space rather than on movement direction alone. However, it should be noted that movement type was associated with different motivational goals across experiments. Accordingly, in experiment 1, extension and flexion were associated with avoidance of a threatening and approach of a non-threatening stimulus, whereas in experiment 2, both movements were performed toward neutral, inanimate objects. Thus, movement type was not identical across experiments and was coupled to affective action goals in experiment 1 but mainly reflects kinematic variation in a neutral context in experiment 2. These differences should be considered possible explanations rather than evidence that the higher-order properties of the stimuli caused the different patterns of tactile suppression. Future studies should disentangle the contributions of stimulus properties, affective context, and movement kinematics to tactile suppression in a systematic manner.

While failure to effectively process movement-related tactile feedback under threat may place the organism at risk, misjudging the extent of the threat may result in hypersensitivity to stimuli or events that do not pose harm. Research on clinical populations point to altered sensorimotor processing for fear- or phobia-inducing contexts.^31^^,^^49^ VR-based treatment approaches make it possible for patients to undergo exposure therapy in an immersive environment.^50^^,^^51^^,^^52^ Our findings may provide insights into the mechanisms that could contribute to tactile suppression under threat and have the potential to inform the development of novel approaches for treatment strategies in these populations. In line with this, haptic user interfaces integrating touch experience (e.g., tactile augmentation) into treatment approaches have shown improved treatment outcomes with experience of coming into contact with fear-inducing stimuli.^51^

### Limitations of the study

Our study has some limitations. First, although the immersive nature of the experiment was supported by participants’ emotional reactions to the spider and rabbit stimuli, there was no physical contact with the stimuli. In real-world settings, moving the arm toward a stimulus often serves the purpose of making contact with it. Therefore, while the absence of tactile feedback was necessary to ensure comparability across conditions in our study, future research should investigate how tactile sensitivity during movement is influenced by actual contact with stimuli.^53^

A second limitation concerns the coupling of affective stimulus and movement kinematics. In experiment 1, approach movements were consistently performed toward the non-threatening stimulus and avoidance movements away from the threatening stimulus. We chose this design to approximate ecologically valid action tendencies (i.e., approaching non-threatening and avoiding threatening stimuli). However, our design did not fully dissociate affective stimulus with movement kinematics. As a result, the observed effects reflect the combined influence of affective context and action goals, and did not address the role of movement kinematics per se. Future studies employing fully crossed designs will be necessary to fully disentangle the relative contributions of affective stimulus properties and movement kinematics.

Additionally, our results showed that tactile suppression differed by movement type in experiment 1, with arm extension leading to greater suppression than arm flexion. To examine whether this pattern could be explained by movement kinematics alone, we conducted a control experiment in which participants extended and flexed their arms toward geometric objects. In this experiment, no reliable differences in tactile suppression between movements were observed. Importantly, experiment 2 was not designed as a direct extension of experiment 1 but as a control to isolate the likely contribution of movement kinematics in the absence of affective context. As such, the movements in experiment 2 did not involve directly comparable arm extension and flexion conditions as in experiment 1, limiting statistical comparability across experiments. Future work using fully crossed designs could further disentangle the relative contributions of movement kinematics, motor predispositions and affective context.

## Resource availability

### Lead contact

Requests for further information and resources should be directed to and will be fulfilled by the lead contact, Belkis Ezgi Arikan (arikan.ezgi@gmail.com).

### Materials availability

This study did not generate new unique reagents or materials.

### Data and code availability

• Data: De-identified human behavioral data have been deposited at Open Science Framework and are publicly available as of the date of publication. Accession link is listed in the key resources table.
• Code: This paper does not report original code.
• Additional information: Any additional information required to reanalyze the data reported in this paper is available from the lead contact upon request.

## Acknowledgments

This study is funded by the 10.13039/501100001659German Research Foundation (Deutsche Forschungsgemeinschaft, DFG) (SFB/TRR 135 TP A3, project no. 222641018) and supported by the 10.13039/501100001659DFG under Germany’s Excellence Strategy (EXC 3066/1 “The Adaptive Mind,” project no. 533717223).

## Author contributions

Conceptualization, B.E.A., M.M., and K.F.; methodology, B.E.A., M.M., and K.F.; investigation, B.E.A. and M.M.; writing – original draft, B.E.A. and M.M.; writing – review and editing, B.E.A., M.M., and K.F.; funding acquisition, K.F.; resources, K.F.; supervision, B.E.A., M.M., and K.F.

## Declaration of interests

The authors declare no competing interests.

## Declaration of generative AI and AI-assisted technologies in the manuscript preparation process

During the preparation of this work the corresponding author used Wordtune and ChatGPT in order to improve the clarity of some sentences. After using this tool, the author reviewed and edited the content as needed and takes full responsibility for the content of the publication.

## STAR★Methods

### Key resources table

**Table:** 

REAGENT or RESOURCE	SOURCE	IDENTIFIER
**Deposited data**		

Behavioral and kinematic data	This study	Database: https://osf.io/km57b/overview

**Software and algorithms**		

GPower Release 3.1.9.7	Faul et al.^54^^,^^55^	www.psychologie.hhu.de
R Studio version 1.4.1717	RStudio Team	www.rstudio.com
Quickpsy	Linares & López-Moliner^56^	https://cran.r-project.org/web/packages/quickpsy/quickpsy.pdf
Unity version 2019.4.24f1	Unity Technologies Inc., San Francisco, CA, USA	https://unity.com/unity-hub
SteamVR (version 1.20.1)	Valve Corporation, Bellevue, Washington, USA	https://store.steampowered.com/app/250820/SteamVR/

**Equipment**		

Alienware desktop PC (Intel Core i9-7980XE CPU at 2.6 GHz, 32 GB RAM, Dual NVidia GeForce GTX1080 Ti GPU)	Dell, Round Rock, Texas, USA	No longer sold
HTC Vive Pro Eye virtual head-mounted-display	HTC Corp., Xindian, New Taipei, Taiwan	https://www.vive.com/sea/product/vive-pro-eye/overview/
HTC Vive Pro controller	HTC Corp., Xindian, New Taipei, Taiwan	https://www.vive.com/sea/product/vive-pro-eye/overview/

### Experimental model and study participant details

#### Experiment 1

43 naive participants (27 females and 16 males, mean ± SD = 22.38 ± 2.68 years old, age data missing from one participant). We aimed for a minimum sample size of *N* = 36 based on an *a-priori* power analysis using G∗Power3.181 (Faul et al., 2009) to test the differences across four dependent group means using an F-test for a medium effect size of interest (*f* = 0.25) with an alpha of 0.05 to achieve a power of 0.95. The participants were right-handed as confirmed by the Edinburgh Handedness Inventory (Oldfield, 1971; mean ± SD = 86.93 ± 18.26, handedness data from one participant is missing). They reported no neurological and/or psychiatric conditions and had normal or corrected-to-normal vision. All participants gave written informed consent in accordance with institutional regulations of Justus Liebig University Giessen (Certificate # 2019-0003) and the Declaration of Helsinki (2013, except for pre-registration).

#### Experiment 2

For Experiment 2, we aimed for a similar sample size as estimated for Experiment 1. In total, 35 naive participants (22 females and 13 males, mean ± SD = 23.17 ± 2.73 years old) took part in Experiment 2. Given the within-subject design with repeated measurements across conditions, this sample size was considered sufficient for the planned analyses.

The participants were right-handed as confirmed by the Edinburgh Handedness Inventory (mean ± SD = 82.99 ± 18.75). They reported no neurological and/or psychiatric conditions and had normal or corrected-to-normal vision. All participants gave written informed consent in accordance with institutional regulations of Justus Liebig University Giessen (Certificate # 2019-0003) and the Declaration of Helsinki (2013, except for pre-registration).

### Method details

#### Experiment 1

##### Apparatus and environment

An HTC Vive Pro Eye virtual head-mounted-display (HMD; HTC Corp., Xindian, New Taipei, Taiwan) was used to present the visual stimuli. The HMD has a field of view that extends approximately ±110° diagonally. The HMD screen has a 1400 × 1600 pixel resolution per eye and a 90 Hz refresh rate. Participants held an HTC Vive Pro controller (“VR Controller”) in their right hand, and the position of the participant’s hand was continuously recorded at 90 Hz. We used the integrated haptic actuator in the VR Controller to present the vibration stimuli to the participant’s right hand. Visual stimuli were created in Unity (version 2019.4.24f1; Unity Technologies Inc., San Francisco, CA, USA) and displayed via SteamVR (version 1.20.1). The experiment was run on an Alienware desktop PC (Intel Core i9-7980XE CPU at 2.6 GHz, 32 GB RAM, Dual NVidia GeForce GTX1080 Ti GPU). The projection was stereoscopic and was actively linked to the position of the participant’s head thus providing motion parallax. Auditory pink noise was played over the HMD’s headphones to ensure that participants could not hear the noise arising from the haptic actuator.

The participants viewed a virtual environment that consisted of four walls which displayed a material consisting of a high resolution seamless repeating image of a forest. The ceiling displayed a material consisting of a seamless repeating sky texture, and the floor surface displayed a seamless repeating grass texture (see Figure 1). The room was 18 m wide, 30 m long, and the ceiling was 15 m tall to create a large forest-like space.

The participant’s right hand, which held the VR Controller, was displayed in the virtual environment as a hand wearing a red leather glove (Figures 1A and 1B). A sphere, referred to as the trial starter sphere was located −13.2 m from the center of the room (1.8 m from the back wall). It had a diameter of 0.36 m and the center was 2.4 m above the ground. At the start of the experiment the participant adjusted their position until they could comfortably place their right hand into the sphere without having to move their feet and faced the center of the environment. The participants were simulated 1m taller in the virtual environment than their real height. This was due to reports during an initial test prior to the experiment that participants felt short in the environment. Therefore, the trial starter sphere being 2.4 m above the ground would be similar to being 1.4 m above the ground if the participant was not displaced.

At the start of the experiment a set of general instructions were displayed on a gray rectangle, referred to as the instruction panel, that was 3 m × 2 m and located roughly 3 m in front of the participant (see Figure 5B). Following each trial, the participants saw a question panel asking if they had felt a vibration. The panel was 10 m × 5 m and was located 15m in front of the participant. The participant responded to the question by sticking their right hand into one of two cubes that appeared. One cube had a material that contained the word “Ja” (Yes) and one had a material with the word “Nein” (No). The cubes were both 0.4 m3, with the center of each cube 1.5m above the ground and roughly 1m in front of the participants. The yes cube was 0.4 m to the left and the no cube was 0.4 m to the right of the trial starter sphere.

On each trial the participants could see one of three stimuli, a baseline stimulus, the threatening stimulus, or the non-threatening stimulus (See Figure 2).

Baseline stimulus: The baseline stimulus was a black sphere. It occupied the same location and dimensions as the trial starter sphere. The baseline stimulus contained an invisible sphere collider which could detect collisions from the virtual controller. The collider was the same dimension as the baseline stimulus.

Threatening stimulus: The threatening stimulus, referred to as the spider was a virtual spider object. The spider was roughly 0.2 m (width) x 0.12 (height) x 0.25 (length) with the exception of the tips of its legs. An invisible collider object also occupied these dimensions with the exception of the tips of the legs. The spider was 1.97 m from the ground. It had a brown hair texture and looped through an animation of it wiggling legs as if it were moving.

Non-threatening stimulus: The non-threatening stimulus, referred to as the rabbit was a virtual rabbit object. The rabbit was roughly 0.2 m (width) x 0.12 (height) x 0.25 (length), excluding the ears. An invisible collider object also occupied these dimensions with the exception of the ears. The rabbit was 1.97 m from the ground. It had a brown fur texture and looped through an animation of it pretending to eat grass.

The threatening and non-threatening stimuli could be near to or far from the participant, depending on the movement they had to perform (extension or flexion). When near, the object was located at −13.35 m and so roughly 0.25m in front of the participant. The object was also offset by 0.10m to the left of the center of the environment. When the object was far from the participant, it was located at −13.05 m so roughly 0.55 m in front of the participant. It was also offset by 0.10 m to the right. The offsets were done so that the participant did not have to reach over the spider object when it was near to them as it was a threatening stimulus.

Following the methods of McManus et al.,^40^ we probed tactile sensitivity by delivering brief vibrations to the palm of the right hand via the VR controller. The vibrations do not correspond to real physical vibrations, but are defined by an arbitrary number between 0 and 1 (in arbitrary units, au). The vibration was presented for 50 ms at 250 Hz at one of eight intensities (0.00099, 0.0033, 0.0066, 0.0099, 0.033, 0.066, 0.099, 0.33 arbitrary units) plus a no-vibration condition. McManus et al.^40^ found that the lowest possible intensity the VR controller could produce was 0.0009 au and so it was the lowest one that we chose to present.

In order for the vibration (or no vibration) to occur on a specific trial certain criteria had to be met; otherwise, the vibration (or no vibration) would not occur and the trial would be a miss trial. During a trial with the threatening or non-threatening stimulus, the participant had to move their right hand faster than 0.3 m/s with 0.1 m/s of that speed needing to be a velocity downwards. This movement speed had to occur more than 10 frames from the start of the trial. Once these criteria were achieved the vibration condition occurred on the next frame. For the baseline condition the vibration condition occurred after 100 ms from the start of the trial and only if the participants hand was inside the black sphere.

#### General procedure

The participants performed a dynamic arm movement where they reached out to touch the non-threatening rabbit stimulus or avoided touching the threatening spider stimulus. In addition, they performed a tactile detection task in which they had to judge whether or not a vibration had been delivered during the trial. The experiment consisted of three experimental conditions. In the threatening stimulus condition, the trial started as soon as the trial starter sphere disappeared and the spider object appeared either near or far from the participant. The participant then had 150 ms to reach with their right hand to the opposite location of the stimulus. For example, if the spider was in the near position, the participant had to reach to the far position. At the far location, there was an invisible collider object which was roughly the same size as the spider object. In the non-threatening stimulus condition, when the trial started and the trial starter sphere disappeared, the rabbit object appeared either near or far from the participant. The participant then had 150 ms to reach with their right hand and touch the rabbit. Finally, in a baseline condition, when the participant started a new trial, the trial starter sphere would turn black indicating the baseline task had started. During this task, the participant had to keep their hand inside the sphere and hold it still. We recorded the position of the hand along each axis-x (left and right), y (up and down) and z (back and forth) on every frame of a trial (every 11 ms).

Upon arrival at the VR lab, participants received a brief explanation of the task, including information that a spider stimulus would be presented in the VR environment. To ensure no participants with arachnophobia were included—despite this being specified in the screening criteria—a picture of the spider was shown beforehand. After confirming eligibility and obtaining informed consent, participants were given detailed instructions on the task, first verbally and then in written form (see below).

Following this, participants were instructed on how to properly wear and adjust the VR headset and how to operate the VR controller. Once the headset was in place, they completed an eye-tracking calibration to ensure correct headset positioning and proper interpupillary distance.

The experiment then began, and participants were asked to position themselves comfortably so they could place their virtual hand into the trial starter sphere. Each trial began with the participant placing their hand in the sphere and pulling the trigger button. They then viewed a short version of the instructions displayed on the Instruction Panel in the headset. Following the written instructions, the participant practiced the tasks before beginning with the actual experiment. In the first set of practice trials, participants practiced starting a trial and moving their right hand to one of the two different distances, near or far, or performing the baseline condition. When moving to the near or far location, the position was indicated using a box object that was roughly the size of the collider of the spider and the rabbit object. The participant had 150 ms to place their hand in the correct location. No vibrations were delivered during this phase and the participant was not asked to report if they felt a vibration. The participant performed 10 trials, 4 for each of the near and far conditions and 2 for the baseline. After the practice trials, the participants performed 10 more trials of the same task but the boxes were now invisible. Once they completed all 20 trials, the participant started the next phase of the practice which was indicated by a new set of instruction slides. The task was the same as before except that the boxes were replaced with the rabbit and spider objects. After completing 5 trials (1 of each task: baseline, spider extend, spider flex, rabbit extend, rabbit flex) a new set of instruction slides appeared informing the participant that a vibration might now be delivered during the trial. The participant then performed the same task but a vibration of 0.3 au occurred on half of the trials. After each trial the participant reported if they felt a vibration or not. After completing 10 trials (2 of each task) an instruction panel reappeared and informed the participant that the practice was ended. After pulling the trigger button the instruction panel disappeared and the trial starter sphere appeared. The participant then started with the actual experiment (see Video S1 for an example of experimental trials). They were instructed to move as quickly but accurately as possible.

Each condition (baseline, spider-extend, spider-flex, rabbit-extend, rabbit-flex) was presented 54 times (9 vibration conditions presented 6 times per condition). This resulted in a total of 270 trials per participant. The order of the trials was randomized across participants. If a mistrial occurred, the participant had to repeat the trial. A mistrial occurred during the conditions involving movement if the participant took longer than 150 frames to move to the correct location or if they hit the spider object. For the baseline condition, a mistrial occurred if the participant moved their hand outside of the black sphere. The occurrence of a mistrial was indicated by a deep sounding beep.

After the VR task, the participants filled out questionnaires regarding the experimental stimuli, state and trait anxiety and their judgments of task demand under different conditions (see Self report measures). The entire procedure took approximately 1 h. The participants could take a break at any time.

Participants 36–43: Prior to completing data collection (after participant 35), we observed that detection thresholds in the baseline condition were slightly higher than expected compared to other experiments in our lab using similar equipment and stimulus intensities e.g.,^7^^,^^40^. We suspected that this was due to the interleaving of the baseline condition with all other movement conditions. We therefore presented the baseline trials in a separate block for the final seven participants. We included the difference in presentation as a fixed effect of no interest in our analyses. As this did not seem to drive detection performance (see Results), we used all the data for the correlation analyses and linear mixed model analyses.

#### Self report measures

We used the self-assessment manikin scale (SAM^57^), a pictorial, non-verbal questionnaire that quantifies emotional reactions toward stimuli. We obtained valence (1 = unpleasant to 9 = pleasant) and arousal (1 = calm to 9 = excited) dimensions for the spider and rabbit stimuli. Participants also rated their anxiety and disgust in encountering the images using a likert-type scale (e.g., for anxiety 1 = not anxious at all, 7 = extremely anxious). To examine the potential role of participant’s state and trait level anxiety on their detection performance, we administered the State-Trait-Anxiety Index (STAI^58^), a self-report questionnaire that quantifies anxiety and depression. The trait level assesses emotionality, worry, euthymia and dysthymia, whereas the state level measures transient and situational anxiety. All items were quantified in a likert scale ranging from 1 (= almost never) to 4 (= almost always). Finally, we administered German translation of the NASA Task Load Index^59^^,^^60^ to assess potential differences in task demand. This questionnaire consists of six subscales measuring different aspects of task demand: Mental demand, physical demand, temporal demand, effort, performance and frustration load. We used the index to investigate potential differences in task demand for the baseline, spider and rabbit stimuli. We calculated a global task load score across all subscales for each stimulus ranging between 0% and 100% with high numbers indicating high task load. Due to experimenter error, a small number of participants (*N* = 6) did not complete all questionnaire items, resulting in a slightly reduced sample sizes for the subjective ratings compared to the threshold measures.

#### Experiment 2

##### Apparatus and environment

The apparatus and stimuli were identical to Experiment 1 except for the following. First, the threatening spider and the non-threatening rabbit were replaced with boxes that were the same gray color as the trial starter sphere (0.15 wide x 0.1 m high x 0.3 m long). The boxes were rotated by 90° such that the longest side was facing the participant. Second, we included three conditions: extension, flexion and baseline (see Figure 7). And third, valence, arousal, anxiety and disgust ratings as well as STAI-state and trait questions were not administered.

##### General procedure

The procedure was identical to the first experiment with the following exceptions. First, the participants did not valence, arousal and anxiety ratings as well as STAI state and trait anxiety questionnaires were not administered. Second, each participant completed 10 practice trials where the box was shown, followed by another 10 practice trials (4 for extension, 4 for flexion, 2 for baseline) where a vibration stimulus with a strength of 0.3au occurred on half of the trials. Following these trials, the instruction panel appeared informing the participant that the actual experiment would now start. Third, each of the three conditions (extend, flex, baseline) occurred 54 times (9 vibration conditions repeated 6 times per condition) for a total of 162 trials with condition order interleaved. The criteria for mistrials and inducing the vibration stimulus were similar to the first experiment for the baseline condition. For the movement conditions, a mistrial occurred if the participant did not collide with the box within 150 frames from the start of the trial. The entire experiment took ∼45 min. The participants could take a break at any time.

### Quantification and statistical analysis

#### Experiment 1

##### Movement kinematics

Using R studio (RStudio 2021.09.1) we plotted the movement kinematics of the hand along each axis (x left and right, y up and down, and z back and forth). First, all mistrials were removed. Then, for each condition, we determined the median frame on which the vibration and collision occurred along with the standard deviation. The median frame the vibration occurred for each condition was: baseline 101 (SD = 0), spider-extend 47 (SD = 14.72), and spider-flex 50 (SD = 23.0), rabbit-extend 47 (SD = 13.69), rabbit-flex 44 (SD = 15.39). The median frame the collision occurred for each condition was: baseline 251 (SD = 31.26), spider-extend 81.75 (SD = 19.73), spider-flex 99.25 (SD = 35.83), rabbit-extend 80.25 (SD = 11.30), and rabbit-flex 71.25 (SD = 11.39).

Then, for each condition, participant, and frame we calculated the average position of the hand and standard deviation of the position of the hand along each axis. We then averaged across participants to get the overall average position of the hand and standard deviation for each frame in each condition for each axis. The frames were converted into time by first dividing the frame number by 90, then multiplying by 1000 to get milliseconds. When plotting the occurrence of the vibration and collision the average for each object was used (i.e., spider, rabbit, baseline, see Figure 6).

##### Calculating movement speed for threatening and non-threatening stimuli

The speed of the hand was determined as the speed of the hand at the time of vibration. We calculated the average speed of the hand for each person and movement condition by averaging over the trials in that condition. Therefore, each participant had four speeds: spider-flex, spider-extend, rabbit-flex, rabbit-extend.

We subjected the data to statistical analyses at the group level. All analyses were implemented in R Studio version 4.4.2.^61^ To test differences across conditions, we implemented linear mixed models (LMMs) using the lmerTest package in R.^62^ The LMMs were fitted using the restricted maximum likelihood estimation. For all LMMs fixed effect terms were estimated with the anova function which performs an F-test on the fixed effects using the Satterthwaite approximation.^63^ For post-hoc pairwise comparisons and estimated marginal means, we used the emmeans package.^64^ For multiple pairwise comparisons, we used Bonferroni adjustment, when necessary. In addition, we computed 95% confidence intervals for the fixed effect coefficients using the confint function. Effect sizes were calculated using the effect size package in R as Cohen’s *d*.^65^

##### Tactile perception

To examine potential differences in tactile perception across conditions, we estimated the detection thresholds for each participant and condition. The data for each condition and participant was fit using R Studio (RStudio Version 1.4.1717) package quickpsy,^56^ using a Weibull distribution and optimized via the Differential Evolution algorithm.^66^ For this algorithm we had to provide upper and lower bounds for the parameters (means and standard deviations). For the upper and lower mean parameters, we chose the upper and lower vibration capabilities of the VR Controller which were 0 (no vibration) and 1 (maximum possible vibration magnitude). For the upper and lower standard deviations, we chose 0 and 2 as these parameters are well-above any values expected from the detection thresholds. The 50% threshold of each psychometric function was used as the detection threshold. This resulted in five detection thresholds per participant for a total of 215 data points (43 participants x five thresholds).

An outlier analysis was then performed on the detection thresholds. A significant outlier was defined as any point that fell outside of the 1st or 3rd interquartile range by three times the interquartile range. No outliers were removed.

Using the estimated thresholds, we conducted two LMMs. The first LMM tested the effect of movement on the detection thresholds and included the conditions baseline, spider-extend, spider-flex rabbit-extend, rabbit-flex, condition randomization (baseline interleaved vs. baseline separate) as a fixed effect and participant as a random effect term. The second LMM tested whether affective stimuli (threatening spider vs. non-threatening rabbit) and movement type (extension vs. flexion) affected detection thresholds. The fixed effects terms for this LMM were affective stimulus, movement type and their interaction, and condition randomization as a fixed effect of no interest. The random effect term was participant. In addition, as tactile suppression can be modulated by movement speed^67^, but see^53^, we included each participant’s average movement speed at the time of vibration in each condition as a covariate in the latter analysis.

We also tested whether the spider stimulus elicited stronger affective responses than the rabbit stimulus. We conducted separate LMMs on the affective ratings (valence, arousal, anxiety, disgust) with affective stimuli (threatening spider vs. non-threatening rabbit) and condition randomization (baseline interleaved vs. baseline separate) as fixed effects and participant as random effect.

In addition, we implemented Spearman correlations to test potential links between the following measures: First, we examined the relationship between emotional responses to the spider and rabbit stimuli defined as valence, arousal ratings for each stimulus and participant’s detection thresholds during different movements. Second, we tested whether participant’s state- and trait anxiety measures correlated with their detection thresholds as a function of affective stimulus and movement type. Finally, a third set of correlations addressed the potential relationship between cognitive task load and detection performance as a function of affective stimulus and movement type. For each question of interest, we corrected the *p*-values for the number of correlations using Bonferroni correction.

##### Response bias

To assess potential response bias in the detection task, we analyzed false alarm rates, defined as the proportion of trials in which participants reported perceiving a vibration when no vibration was presented. For each participant and condition, false alarm rates were calculated as the proportion of “yes” responses on trials without a vibration stimulation. We conducted a LMM on the false alarm rates with condition (baseline, spider-extend, spider-flex rabbit-extend, rabbit-flex), condition randomization (baseline interleaved vs. baseline separate) as a fixed effect and participant as a random effect term.

#### Experiment 2

##### Tactile perception

Individual data from each participant and condition were fitted as in Experiment 1. Data from one participant was removed as they could not reliably detect the highest vibration intensity. This resulted in data from 34 participants being used to estimate the detection thresholds. After calculating the thresholds, we performed the same outlier analysis as in Experiment 1 where a data point was removed if it fell outside of the 1st or 3rd interquartile range by 3 times the interquartile range. Accordingly, three data points (2.94% of all data) were removed (two data points from the baseline condition, one data point from the extend condition).

Group-level analyses were implemented using the same software and functions. We conducted a LMM to test the effect of movement on the detection thresholds by including the fixed effects terms movement (extend, flex, baseline) and the random effect term participant. We conducted a second LMM in which we included average movement speed as a covariate. As the frequentist analyses did not reveal evidence for an effect of movement (see Results), we complemented these analyses with a Bayesian approach.^68^ Using the package BayesFactor^69^ in RStudio, we quantified the evidence for the (alternative) hypothesis against the null hypothesis, expressed as the Bayes Factor (BF) of the alternative hypothesis (H1) over the null hypothesis (H0) (BF10). A Bayesian paired-samples *t* test compared detection thresholds between movements (extend vs. flex), with a Cauchy prior on the standardized effect size centered at 0 and scale parameter r = 0.707. To qualitatively evaluate the strength of evidence given by BF10, we used the adjusted Jeffreys Scale of Evidence^70^:

BF10 > 100: Extreme evidence for H1. BF10 = 30–100: Very strong evidence for H1. BF10 = 10–30: Strong evidence for H1. BF10 = 3–10: Moderate evidence for H1. BF10 = 1–3: Anecdotal evidence for H1. BF10 = 1/3–1: Anecdotal evidence for H0. BF10 = 1/10–1/3: Moderate evidence for H0. BF10 = 1/30–1/10: Strong evidence for H0. BF10 = 1/100–1/30: very strong evidence for H0. BF10 < 1/100: Extreme evidence for H0.

Moreover, we conducted correlation analyses between task load and detection thresholds.

The hand movement kinematics were calculated similarly to Experiment 1 (see Figure 9).

##### Response bias

A LMM on the false alarm rates with condition (baseline, extend, flex) as a fixed effect and participant as a random effect term was conducted to test if false alarm rates differed between conditions.

## Supplemental information

Document S1. Figures S1–S7 and Tables S1–S7

Video S1. Examples of experimental trials in Experiment 1 The video shows representative trials from the different experimental conditions in the virtual environment, presented in the following order: rabbit-extend, spider-flex, baseline, rabbit-flex, baseline, and spider-extend
