# Supplementary material for: Avoiding spiders: Affective context shapes tactile sensitivity during movement
Source: iScience. 2026 Oct 15;29(11):117902. doi: 10.1016/j.isci.2026.117902 (PMC13691314; doi:10.1016/j.isci.2026.117902)
Supplement: Supplementary file 1 [file mmc1.pdf]

iScience, Volume *29*

Supplemental information

Avoiding spiders: Affective context shapes tactile sensitivity during movement

Belkis Ezgi Arikan, Meaghan McManus, and Katja Fiehler

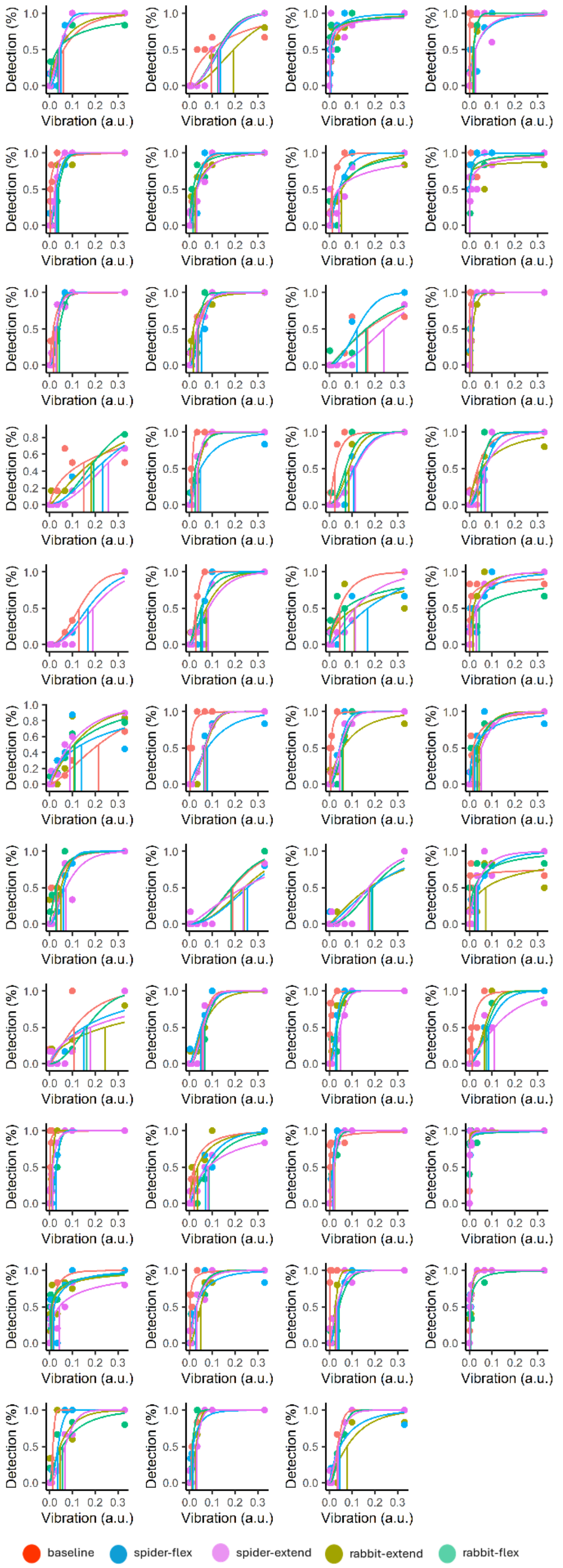

Figure S1. Individual psychometric functions depicting tactile detection as a function of tactile intensity in all conditions in Experiment 1. In line with Fechner's law, the vibrations used in this experiment follow a log scale (plus the no vibration condition). When determining the individual thresholds we included the no-vibration condition and so could not plot the data on a logged scale.

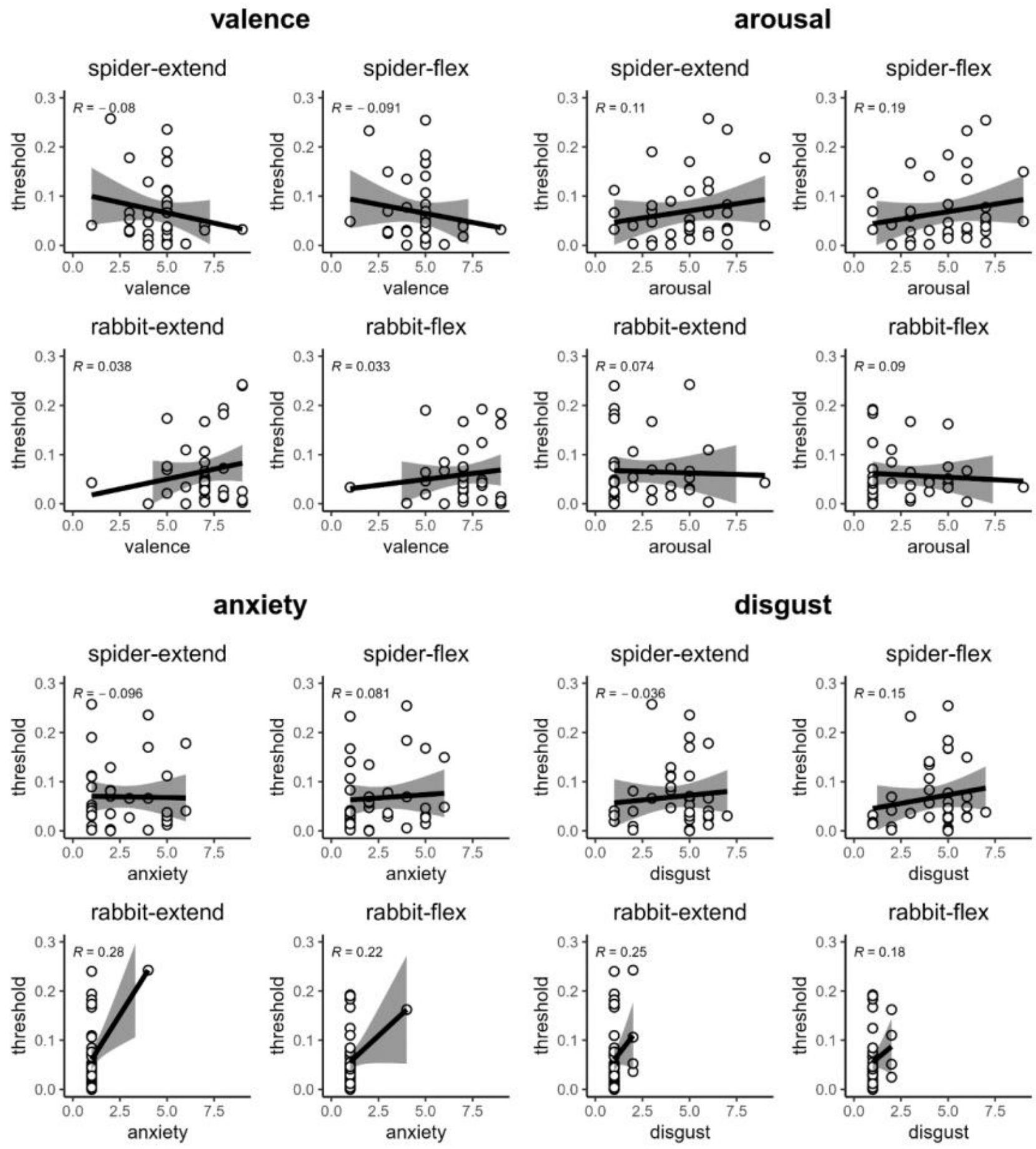

Figure S2. Correlation between detection thresholds and valence, arousal, anxiety and disgust ratings as a function of affective stimuli and movement. Dots represent individual data points (valence: n = 37 in all conditions; arousal: n = 37 in all conditions; anxiety: n = 37 in all conditions; disgust: n = 37 in all conditions). Line and shading represent the best-fitting regression line and its 95% confidence interval, respectively. All $p > .05$ after correction for multiple comparisons.

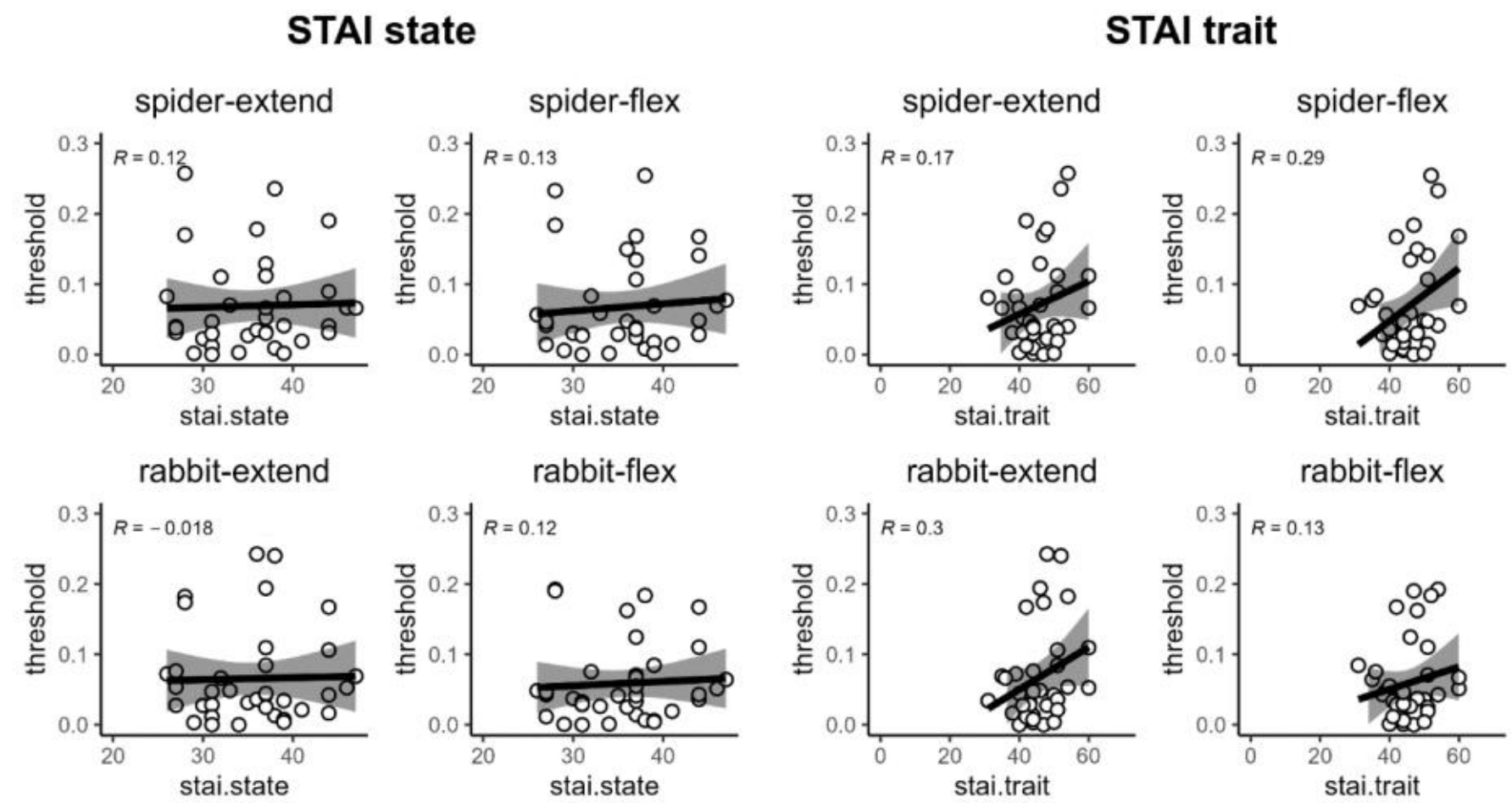

Figure S3. Correlation between detection thresholds and STAI-state and STAI-trait measures as a function of affective stimuli and movement. Dots represent individual data points (STAI state: n = 37 in all conditions; STAI trait: n = 36 in all conditions). Line and shading represent the best-fitting regression line and its 95% confidence interval, respectively. All $p > .05$ after correction for multiple comparisons.

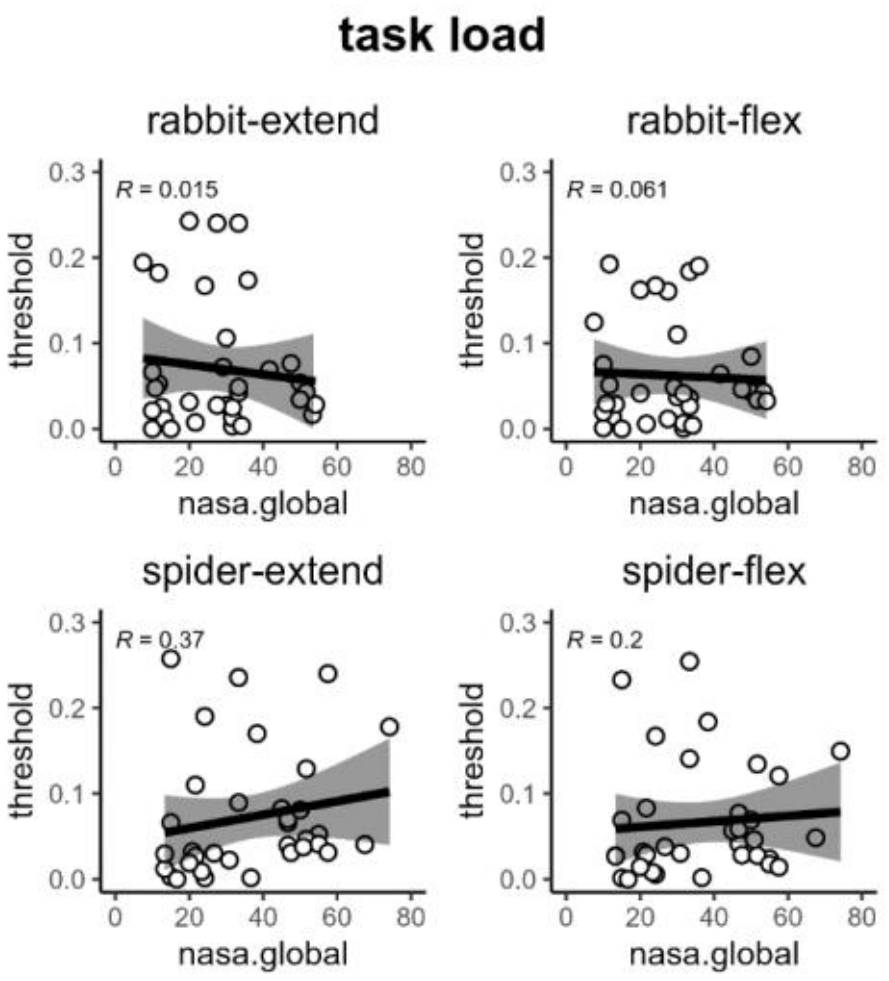

Figure S4. Correlation between detection thresholds and NASA task load ratings as a function of affective stimuli and movement. Dots represent individual data points (n = 34 in all conditions). Line and shading represent the best-fitting regression line and its 95% confidence interval, respectively. All $p > .05$ after correction for multiple comparisons.

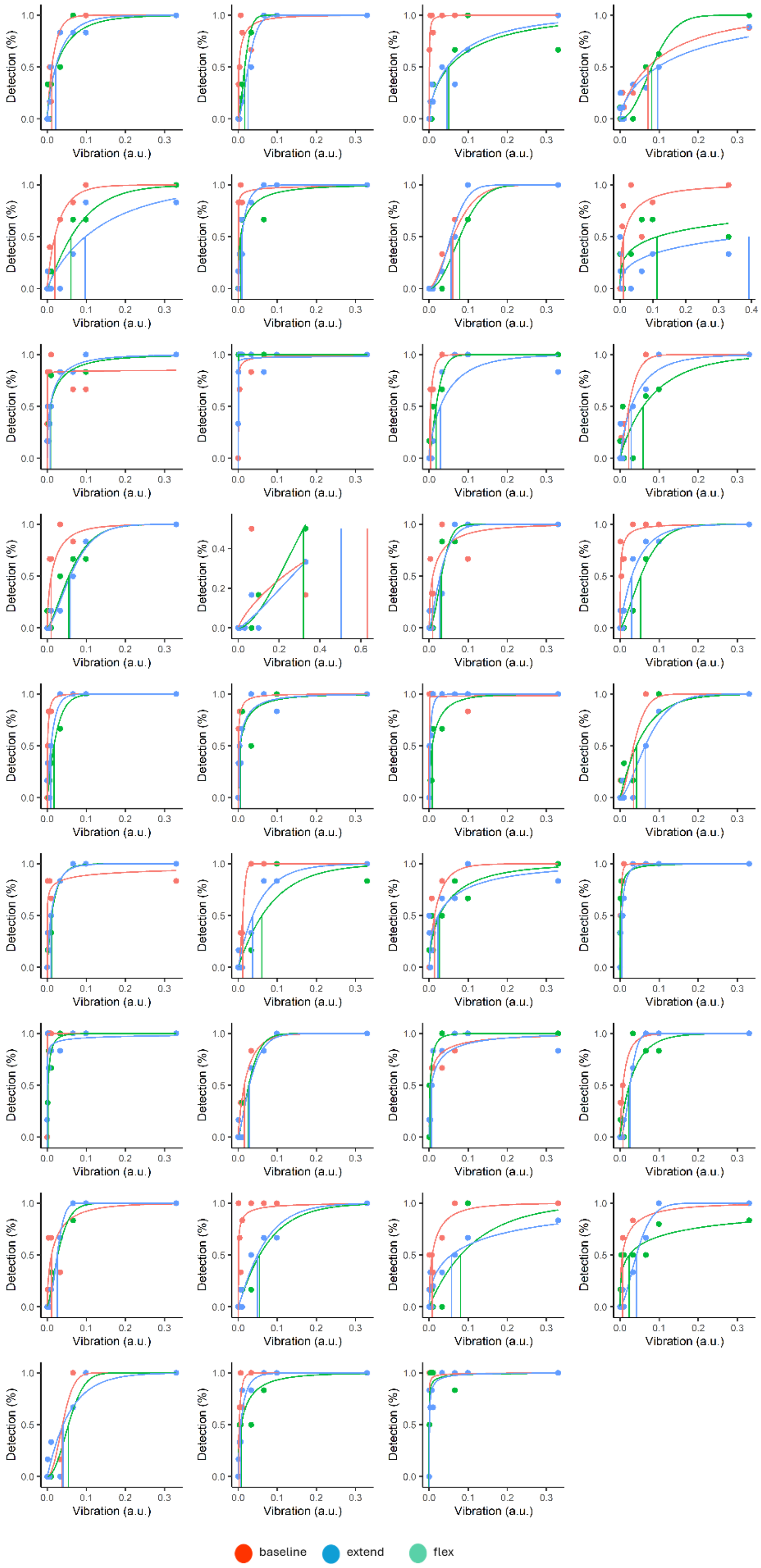

Figure S5. Individual psychometric functions depicting tactile detection as a function of tactile intensity in all conditions in Experiment 2. In line with Fechner's law, the vibrations used in this experiment follow a log scale (plus the no vibration condition). When determining the individual thresholds we included the no-vibration condition and so could not plot the data on a logged scale.

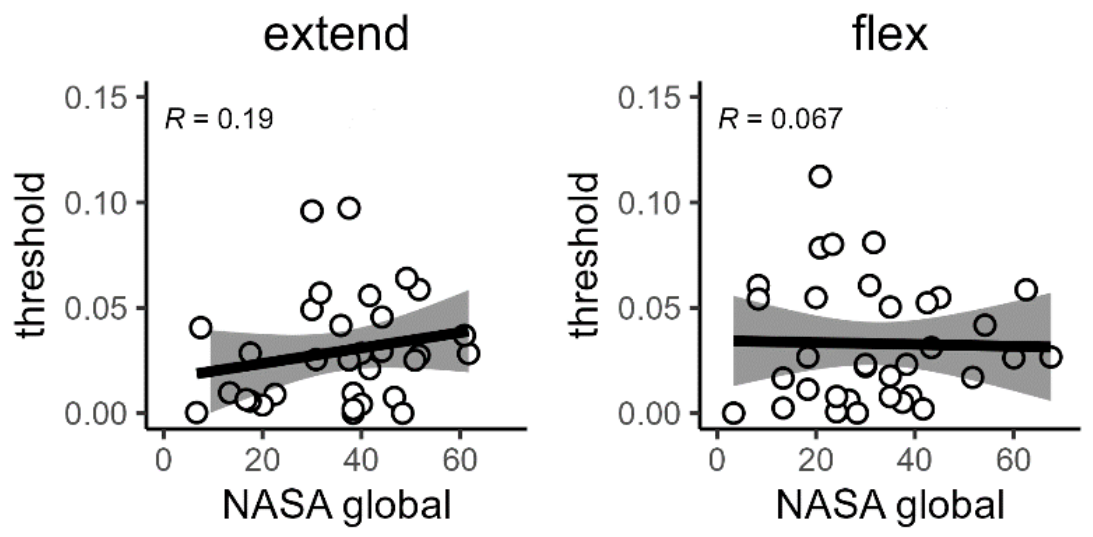

Figure S6. Correlation between detection thresholds and NASA task load ratings as a function of movement type in Experiment 2. Dots represent individual data points (n = 33 in extend and n = 34 in flex). Line and shading represent the best-fitting regression line and its 95% confidence interval, respectively. All *p* > .05 after correction for multiple comparisons.

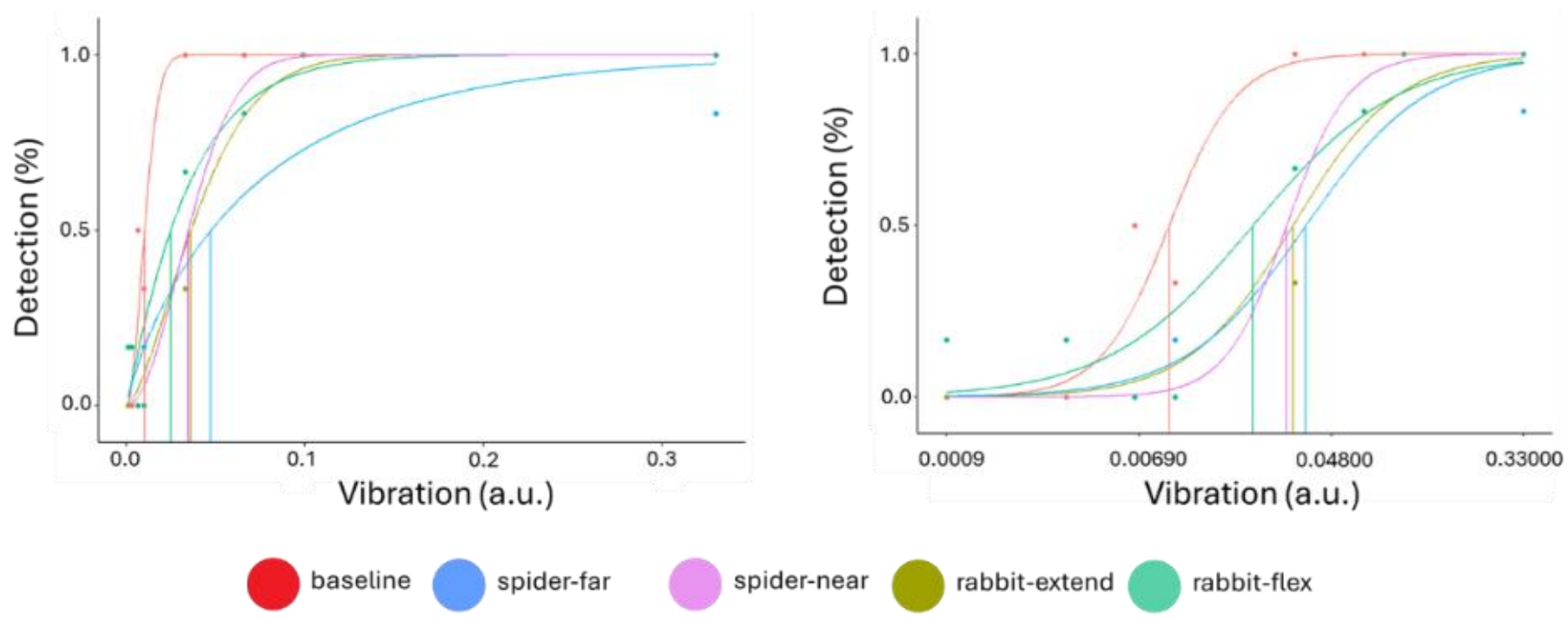

Figure S7. A randomly selected individual's data fitted to the Weibull psychometric function and logistic function. The panel on the left shows the data plotted as in the manuscript. The panel on the right shows the same participant's data plotted on a logged scale, with 0 removed, with a logistic function, and with the x-axis constrained between 0.0009 and 0.33000.

Table S1. Thresholds and deviance score for each participant and condition in Experiment 1. The deviance score is displayed within the brackets. The means, standard deviations, and standard errors for each condition are displayed at the bottom of the table.

participant	baseline	rabbit-extend	rabbit-flex	spider-flex	spider-extend
1	0.060736 (77.0155)	0.042371 (9.2985)	0.036161 (11.3564)	0.048495 (72.1648)	0.040636 (4.7152)
2	0.097279 (6.7301)	0.194029 (2.8619)	0.124521 (3.2265)	0.134468 (3.3626)	0.128968 (3.5746)
3	0.007031 (74.0417)	0.003146 (4.2026)	0.000628 (5.8592)	0.005834 (5.773)	0.001625 (8.5487)
4	0 (5.3209)	0.016037 (1.8732)	0.017817 (3.4991)	0.025378 (7.2514)	0.026947 (9.1265)
5	0.00213 (5.3444)	0.027528 (7.2873)	0.037208 (69.8745)	0.03034 (68.6804)	0.022544 (2.7875)
6	0.029307 (3.609)	0.025374 (4.4916)	0.014446 (3.864)	0.031867 (8.3755)	0.032266 (7.9949)
7	0.010967 (73.0325)	0.05359 (8.7216)	0.0428 (15.5677)	0.041511 (7.1104)	0.039892 (11.9231)
8	0.000482 (365.5876)	0.000031 (217.6235)	0.001074 (290.6907)	0.00163 (212.4794)	0.002944 (288.8278)

9	0.015327<br>(4.8811)	0.031409<br>(1.9109)	0.041442<br>(0.9881)	0.028839<br>(1.609)	0.026972<br>(5.127)
10	0.038304<br>(5.6702)	0.015741<br>(3.0872)	0.035888<br>(6.7746)	0.052017<br>(2.4868)	0.031155<br>(4.3014)
11	0.168022<br>(5.5074)	0.240074<br>(2.5809)	0.160649<br>(15.9075)	0.120707<br>(4.3212)	0.240074<br>(2.5808)
12	0.000496<br>(4.8739)	0.013064<br>(3.7003)	0.00665<br>(3.2394)	0.008172<br>(0.9715)	0.00919<br>(1.2461)
13	0.148675<br>(6.2477)	0.182218<br>(5.4767)	0.192371<br>(2.2793)	0.232906<br>(2.6462)	0.25747<br>(0.8919)
14	0.010187<br>(3.1727)	0.036178<br>(5.3791)	0.024868<br>(8.8183)	0.047328<br>(10.2802)	0.034757<br>(2.424)
15	0.024019<br>(7.0249)	0.084435<br>(3.7757)	0.070667<br>(10.4258)	0.106652<br>(4.7234)	0.112087<br>(1.1005)
16	0.035947<br>(8.7268)	0.052564<br>(11.7476)	0.051262<br>(8.457)	0.069061<br>(2.3849)	0.066292<br>(15.2638)
17	0.129363<br>(0.7503)	0.167151<br>(2.5912)	0.167151<br>(2.5912)	0.167151<br>(2.5912)	0.190118<br>(5.1309)
18	0.027529<br>(1.8635)	0.072269<br>(8.8463)	0.048441<br>(6.4527)	0.056654<br>(4.0347)	0.082544<br>(10.8001)
19	0.04352<br>(6.8549)	0.109508<br>(12.2608)	0.067114<br>(76.4438)	0.167957<br>(5.4966)	0.111766<br>(6.5218)
20	0.001133<br>(9.486)	0.016587<br>(10.4269)	0.042994<br>(3.6749)	0.02833<br>(15.7148)	0.031149<br>(7.6399)
21	0.214765<br>(1.1663)	0.106152<br>(9.5747)	0.110196<br>(7.2902)	0.140782<br>(14.4883)	0.089443<br>(3.0183)
22	0.00346<br>(4.3029)	0.069061<br>(2.3849)	0.064232<br>(0.3448)	0.077184<br>(6.3793)	0.065982<br>(0.2987)
23	0.004494<br>(5.4242)	0.057959<br>(11.6815)	0.056611<br>(4.0159)	0.043716<br>(4.7942)	0.045541<br>(2.5056)
24	0.016111<br>(6.9705)	0.04293<br>(2.814)	0.033649<br>(9.3276)	0.024027<br>(7.2144)	0.052585<br>(6.7028)
25	0.029881<br>(8.4909)	0.04867<br>(138.7035)	0.026351<br>(9.445)	0.058754<br>(0.6162)	0.070269<br>(7.6812)
26	0.190114<br>(5.1305)	0.240041<br>(2.5785)	0.183688<br>(4.5636)	0.254344<br>(2.2005)	0.235549<br>(12.1964)
27	0.190118<br>(5.1309)	0.173629<br>(18.3419)	0.190118<br>(5.1309)	0.183809<br>(19.1549)	0.170056<br>(11.9938)
28	0.000051<br>(3.7056)	0.071423<br>(9.6419)	0.021623<br>(214.0859)	0.038473<br>(3.4083)	0.02942<br>(11.2381)
29	0.107481<br>(80.4434)	0.242515<br>(11.1718)	0.162262<br>(2.4881)	0.149533<br>(15.2213)	0.178015<br>(18.3158)
30	0.054198<br>(4.7848)	0.058418<br>(4.9499)	0.065446<br>(5.4905)	0.048591<br>(14.941)	0.059687<br>(4.0825)
31	0.001324<br>(2.7275)	0.028417<br>(2.9518)	0.032526<br>(7.5503)	0.027802<br>(1.8853)	0.046813<br>(1.355)
32	0.013148<br>(3.9708)	0.066384<br>(5.8797)	0.075316<br>(2.2982)	0.083198<br>(1.103)	0.110104<br>(6.5805)
33	0.00014<br>(9.3537)	0.011395<br>(1.4488)	0.028753<br>(1.5361)	0.027604<br>(1.9248)	0.012157<br>(3.3626)

34	0.019512 (5.2517)	0.034214 (17.0603)	0.084523 (10.5385)	0.069139 (4.2584)	0.080945 (5.8292)
35	0.00047 (5.7233)	0.021296 (1.0888)	0.019312 (2.0913)	0.014603 (5.537)	0.019089 (0.7111)
36	0 (70.436)	0.000043 (2.8791)	0.000013 (141.2845)	0.000048 (6.0826)	0.000197 (3.6106)
37	0.011458 (138.7337)	0.007593 (214.3907)	0.005897 (144.6867)	0.017743 (85.3872)	0.041046 (5.5799)
38	0.001332 (5.3198)	0.047422 (2.0415)	0.029337 (7.832)	0.026886 (15.2512)	0.029679 (74.4442)
39	0.000616 (0)	0.024825 (2.3895)	0.041415 (9.897)	0.038033 (1.0487)	0.030405 (73.3429)
40	0.004952 (0.5304)	0.003761 (2.5849)	0.004091 (5.6556)	0.001965 (2.6475)	0.001792 (1.9118)
41	0.013097 (2.5784)	0.044426 (144.9276)	0.055026 (76.4448)	0.035849 (4.7205)	0.066186 (1.412)
42	0.010288 (1.7489)	0.027629 (68.6232)	0.011597 (4.5833)	0.014266 (71.5627)	0.031409 (68.5915)
43	0.035077 (2.5386)	0.076362 (12.1011)	0.046251 (1.5051)	0.046379 (11.4492)	0.037678 (9.7993)
Mean	0.0412 (24.4234)	0.0672 (23.6826)	0.0589 (28.5599)	0.0658 (17.2961)	0.0696 (17.0951)
Standard Deviation	0.0599 (61.0323)	0.0683 (52.6325)	0.0551 (60.6567)	0.0628 (36.8267)	0.067 (45.9169)
Standard Error	0.0092 (9.4175)	0.0105 (8.1214)	0.0085 (9.3595)	0.0097 (5.6825)	0.0103 (7.0851)

Table S2. Planned post-hoc pairwise comparisons on detection thresholds as a function of condition. Statistics are reported for each item with estimate, standard error (*SE*), degrees of freedom (*df*), t-value (*t*) and Bonferroni-corrected *p*-value (*p*).

Contrast	*Estimate*	*SE*	*df*	*t*	*p*
baseline – spider-extend	-0.028	0.005	168	-6.19	< 0.001
baseline – spider-flex	-0.025	0.005	168	-5.35	< 0.001
baseline – rabbit-extend	-0.026	0.005	168	-5.66	< 0.001
baseline – rabbit-flex	-0.018	0.005	168	-3.85	0.0008

Table S3. Mean ratings (±sd) for the affective stimuli.

	**spider**	**rabbit**
**valence**	4.62 (±1.47)	6.89 (±1.57)
**arousal**	4.86 (±2.10)	2.62 (±2.02)
**anxiety**	2.70 (±1.75)	1.08 (±0.49)
**disgust**	4.19 (±1.66)	1.11 (±0.32)

Table S4. Mean global task load (±sd) for the affective and the baseline stimuli.

Experiment 1			Experiment 2		
**spider**	**rabbit**	**baseline**	**extend**	**flex**	**baseline**
36.7 (±17.2)	28.1 (±14.2)	31.7 (±14.0)	36.8 (±15.7)	31.8 (±15.7)	27.3 (±11.6)

Table S5. Thresholds and deviance score for each participant and condition in Experiment 2. The deviance score is displayed within the brackets. The means, standard deviations, and standard errors for each condition are displayed at the bottom of the table.

participant	baseline	flex	extend
1	0.011682 (4.7233)	0.022162 (12.4351)	0.021128 (5.1293)
2	0.002052 (6.8348)	0.016671 (1.0769)	0.025475 (2.0182)
3	0.000158 (3.8571)	0.050404 (12.7775)	0.04563 (4.4457)
4		0.081003 (68.5017)	0.095982 (76.2996)
5	0.019178 (4.9885)	0.06065 (6.457)	0.097332 (12.0308)
6	0.000009 (3.7454)	0.006235 (4.2183)	0.008968 (71.0362)
7		0.078475 (1.9897)	0.055803 (2.2966)
8	0.010941 (13.7682)	0.112421 (146.4023)	
9	0 (142.9779)	0.008198 (69.5191)	0.00752 (71.9581)
10	0.000034 (5.8516)	0.000019 (136.5364)	0 (141.9401)
11	0.003503 (68.3613)	0.017653 (69.8982)	0.028475 (17.2388)
12	0.022105 (5.1707)	0.058596 (81.2387)	0.028282 (11.6771)
13	0.010223 (12.7258)	0.054891 (69.9164)	0.058718 (2.0891)
14	0.009187 (14.758)	0.031166 (4.7841)	0.027526 (9.2118)
15	0.000896 (6.0844)	0.052434 (3.24)	0.02945 (5.9331)
16	0.000886 (0.8114)	0.01703 (71.0114)	0.009492 (77.382)
17	0.000463 (2.901)	0.005195 (7.7076)	0.004311 (5.8652)
18	0 (4.7985)	0.007876 (8.9495)	0.001951 (3.6315)
19	0.034404 (13.2746)	0.041794 (8.8623)	0.063991 (6.3361)
20	0.000173 (9.0881)	0.011356 (2.9747)	0.009729 (4.6687)

21	0.011395 (1.4488)	0.06057 (13.031)	0.036919 (6.783)
22	0.013697 (6.7819)	0.026239 (73.2369)	0.022648 (217.4488)
23	0.000991 (0.9818)	0.000668 (140.2271)	0.003894 (1.5768)
24	0.0001 (0)	0.00201 (69.7334)	0.000012 (73.0245)
25	0.015371 (4.1756)	0.026702 (3.4079)	0.02856 (8.6318)
26	0.002352 (2.5783)	0.002512 (2.6503)	0.005716 (76.4354)
27	0.007329 (7.4305)	0.023121 (13.173)	0.02519 (1.0924)
28	0.010731 (17.6859)	0.026671 (3.4567)	0.025283 (1.0748)
29	0.00035 (14.2146)	0.054841 (2.6517)	0.049228 (3.5161)
30	0.007814 (73.7353)	0.08017 (12.353)	0.057024 (4.7653)
31	0.005958 (4.2669)	0.023157 (3.3021)	0.041634 (3.7437)
32	0.038769 (4.1831)	0.054198 (4.7848)	0.040623 (15.3795)
33	0.002561 (5.4401)	0.007939 (8.7965)	0.006502 (2.676)
34	0.000004 (4.1817)	0.000041 (9.4631)	0.000332 (4.2808)
Mean	0.0076 (14.7445)	0.033 (33.7872)	0.0292 (28.8369)
Standard Deviation	0.0098 (28.6261)	0.0288 (43.8562)	0.0257 (48.2513)
Standard Error	0.0018 (5.1414)	0.005 (7.6344)	0.0045 (8.5297)

Note: Empty cells represent the data points that were removed during the outlier analysis.

Table S6. Planned post-hoc pairwise comparisons on detection thresholds as a function of condition. Statistics are reported for each item with estimate, standard error (*SE*), degrees of freedom (*df*), t-value (*t*) and Bonferroni-corrected *p*-value (*p*).

Contrast	*Estimate*	*SE*	*df*	*t*	*p*
baseline – extend	-0.02	0.004	63.6	-5.65	< 0.001
baseline – flex	-0.02	0.004	63.4	-6.52	< 0.001
extend – flex	-0.003	0.004	63.2	-0.82	> 0.99

Table S7. Planned post-hoc pairwise comparisons on false alarm rates as a function of condition. Statistics are reported for each item with estimate, standard error (*SE*), degrees of freedom (*df*), t-value (*t*) and Bonferroni-corrected *p*-value (*p*).

Contrast	*Estimate*	*SE*	*df*	*t*	*p*
baseline – extend	-0.05	0.02	68	-2.16	0.1
baseline – flex	-0.05	0.02	68	-2.18	0.1
extend – flex	-0.0003	0.02	68	-0.02	> 0.99
